# Expanding our understanding of digital mental health interventions for Indigenous youth: An updated systematic review

**DOI:** 10.1177/1357633X241239715

**Published:** 2024-04-07

**Authors:** Lydia J Hicks, Elaine Toombs, Jessie Lund, Kristy R Kowatch, Carol Hopkins, Christopher J Mushquash

**Affiliations:** 1 Department of Psychology, 7890 Lakehead University - Thunder Bay Campus, ON, Canada; 2 Dilico Anishinabek Family Care, Fort William First Nation, Canada; 3 Thunderbird Partnership Foundation, Bothwell, ON, Canada; 4 Thunder Bay Regional Health Sciences Centre, ON, Canada; 5 Thunder Bay Regional Health Research Institute, ON, Canada

**Keywords:** Indigenous youth, eHealth, telemental health, digital mental health, internet intervention, telehealth, systematic review

## Abstract

Past research has examined available literature on electronic mental health interventions for Indigenous youth with mental health concerns. However, as there have recently been increases in both the number of studies examining electronic mental health interventions and the need for such interventions (i.e. during periods of pandemic isolation), the present systematic review aims to provide an updated summary of the available peer-reviewed and grey literature on electronic mental health interventions applicable to Indigenous youth. The purpose of this review is to better understand the processes used for electronic mental health intervention development. Among the 48 studies discussed, smoking cessation and suicide were the most commonly targeted mental health concerns in interventions. Text message and smartphone application (app) interventions were the most frequently used delivery methods. Qualitative, quantitative, and/or mixed outcomes were presented in several studies, while other studies outlined intervention development processes or study protocols, indicating high activity in future electronic mental health intervention research. Among the findings, common facilitators included the use of community-based participatory research approaches, representation of culture, and various methods of motivating participant engagement. Meanwhile, common barriers included the lack of necessary resources and limits on the amount of support that online interventions can provide. Considerations regarding the standards and criteria for the development of future electronic mental health interventions for Indigenous youth are offered and future research directions are discussed.

## Introduction

Indigenous youth are exposed to many elements that increase their risk for negative mental health outcomes. Colonial legacies (e.g. assimilation, gendered violence, dispossession of land and resources)^1^ have historically and continually shaped the lives of Indigenous youth. More broadly, colonialism (i.e. the guiding force that created political and economic inequalities) has been discussed as a social determinant of health that has led to various unjust life situations.^2^ For example, Indigenous populations are more likely to experience decreased housing quality,^3^ unstable housing,^4^ and food security,^5^ as well as increased household crowding,^6^ poverty,^7^ substance use,^8^ and sexual violence^9^ than non-Indigenous populations. Indigenous youth, specifically, are overrepresented in child protection and youth justice systems and are more likely to experience neglect and abuse, as well as poor outcomes in areas of health, education, and social development, when compared to non-Indigenous youth.^10^ As such, Indigenous youth have been noted as constituting a ‘high-risk group’ for emotional and psychological difficulties.^10^ With the accumulation and intersection of many of these risk factors,^11^ there is a clear need for interventions that specifically support the mental health of Indigenous youth.

Indigenous youth have experienced barriers to mental health care for decades. Barriers to accessing mental health services for Indigenous young people included inconsistent access to mental health services,^12,13^ lack of awareness of services,^11^ and discomfort in seeking help from services due to concerns about confidentiality, shame, fear, language or literacy difficulties, and racism^14^ (for a review, see Brown et al.^15^). Retaining mental health service providers within rural or remote communities is also difficult,^12^ and this low retention of staff can then lead to higher caseloads for the remaining staff.^16^ As Indigenous communities can be geographically isolated, there are limited options for supervision and training for service providers, which can result in novice practitioners feeling pressured to practice far beyond their capabilities.^17^ During the COVID-19 pandemic, populations who are the most vulnerable (i.e. Indigenous youth) were left behind when it came to accessing the rapidly developing, innovative, wellness and self-care virtual services that were being designed to meet the needs of the general population.^18^ Conventional mental health supports (i.e. face-to-face care) were also no longer able to meet the increased volume of need for support,^19,20^ and in many circumstances, in-person support was no longer an option.^19,20^ Thus, adaptation and transformation of mental health care options were greatly needed,^18^ especially for Indigenous youth who had long been facing such barriers to care.

Electronic mental health (e-MH) interventions have been suggested as a method to circumvent health care disparities, including service costs, accessibility, and stigma-related barriers.^21^ e-MH refers to mental health services and information that are either delivered or enhanced through the Internet and/or other similar types of technology.^22^ A recent meta-analysis found telehealth interventions (including modalities such as video-conferencing and mobile health apps, etc.) to be equally or more clinically effective when compared to usual care.^23^ As well, telehealth did not compromise the effectiveness of care when compared to conventional forms of health service delivery.^23^ Culturally appropriate care can also be easily integrated into e-MH interventions,^24^ making them particularly relevant for use by Indigenous youth who strongly benefit from the inclusion of culture in care.^25^ Telehealth has also been found to enhance culturally appropriate care by allowing for care to be provided within supportive environments, such as Indigenous-run primary healthcare programs, instead of within mainstream healthcare programs, and by reducing burdens associated with traveling and dislocating from community and family.^26^ As well, literature suggests that young people may be more likely to respond to, and prefer, alternative service delivery strategies offered via e-MH interventions (e.g. video conferencing; and Internet-based applications).^27^ In addition to being an option for reducing geographical barriers to care, e-MH interventions may have additional utility as they might help Indigenous youth feel more comfortable and/or motivated to engage in technology-based services.

Some reports have examined the use of e-MH interventions to support the well-being of Indigenous youth during the peak of the COVID-19 pandemic. For example, Walker et al.^28^ examined articles identifying existing and expected mental health outcomes associated with COVID-19 for Indigenous young people and found that, while digital technologies can help build strong cultural identity, enhance connections to community, and improve mental, social, and emotional well-being outcomes, there are still great inequities in accessibility to digital technology for Indigenous young people. The authors advocated for more targeted policy and funding to promote Indigenous young people's access to digital well-being services.^28^ Relatedly, a review of Strudwick et al.^29^ digital interventions to support the mental health of individuals living in Canada during the COVID-19 pandemic briefly highlighted the lack of digital resources available that are specific to Indigenous peoples and communities.^29^ These reviews demonstrate the potential of e-MH interventions for Indigenous youth, but also demonstrate some key barriers in terms of intervention accessibility.

Several reviews have been conducted to examine the impact of e-MH interventions for Indigenous populations more broadly,^30–35^ but there is still a need for more literature pertaining to e-MH services for Indigenous youth specifically,^36^ specifically around the facilitators and barriers to e-MH intervention implementation. Recent efforts to summarize the available e-MH services for Indigenous youth thus far have offered preliminary findings but noted that further research is needed to confirm those findings.^37^ Povey et al.^38^ currently examining more specific aspects of e-MH interventions for Indigenous youth (i.e. best practices for engaging youth in the development and evaluation of e-MH resources), however, an updated review of the broader considerations pertinent to e-MH interventions for Indigenous youth was still needed as (a) the literature on these interventions has increased and (b) the need for these interventions options have also increased throughout the COVID-19 pandemic. As well, as the First Nations Mental Wellness Continuum Framework (FNMWCF)^39^ and the Integrated Life Course and Social Determinants Model of Aboriginal Health (ILCSDAH)^40^ both highlight the importance of interventions that extend beyond the individual and into the family and community interventions to improve health outcomes, this review will also aim to include e-MH interventions that work to improve outcomes for Indigenous youth via the inclusion of family and community members.

### Study purpose

This study aimed to provide an updated systematic review of the literature describing the implementation of e-MH interventions designed for/with Indigenous youth. We sought to capture any new and relevant studies that had been put forth since the previous study^37^ and to summarize relevant characteristics, facilitators (i.e. factors that increase intervention development, implementation, and accessibility), and barriers (i.e. factors that decrease intervention development, implementation, and accessibility) of e-MH interventions that were reported. Throughout the Introduction above, various terms related to e-MH interventions (i.e. telehealth and digital technologies) have been/are used interchangeably. Throughout the rest of this article, we try when possible to be consistent with the term “e-MH intervention,” however, we also use language reflected within individual papers (i.e. “mobile interventions” and “web-based programs”). In doing so, we are aiming to provide the best summary possible of available e-MH interventions, regardless of cross-study differences in terminology.

The present study is also *process-based* rather than *outcome-based*. More specifically, this systematic review was not conducted to thoroughly review aspects of outcome measures (e.g. efficacy for various outcomes). Instead, only basic counts and descriptions of outcomes are reported, and processes used to develop e-MH interventions for Indigenous youth were the focus, as this type of information would be valuable for future researchers, stakeholders, and/or communities looking to develop or adapt e-MH interventions for their own contexts. With this goal in mind, considerations for standards and criteria that can be used to inform new development e-MH interventions for Indigenous youth were provided based on how often various processes occurred in the literature reviewed and how well they addressed known barriers to intervention.

## Method

**Figure 1.**
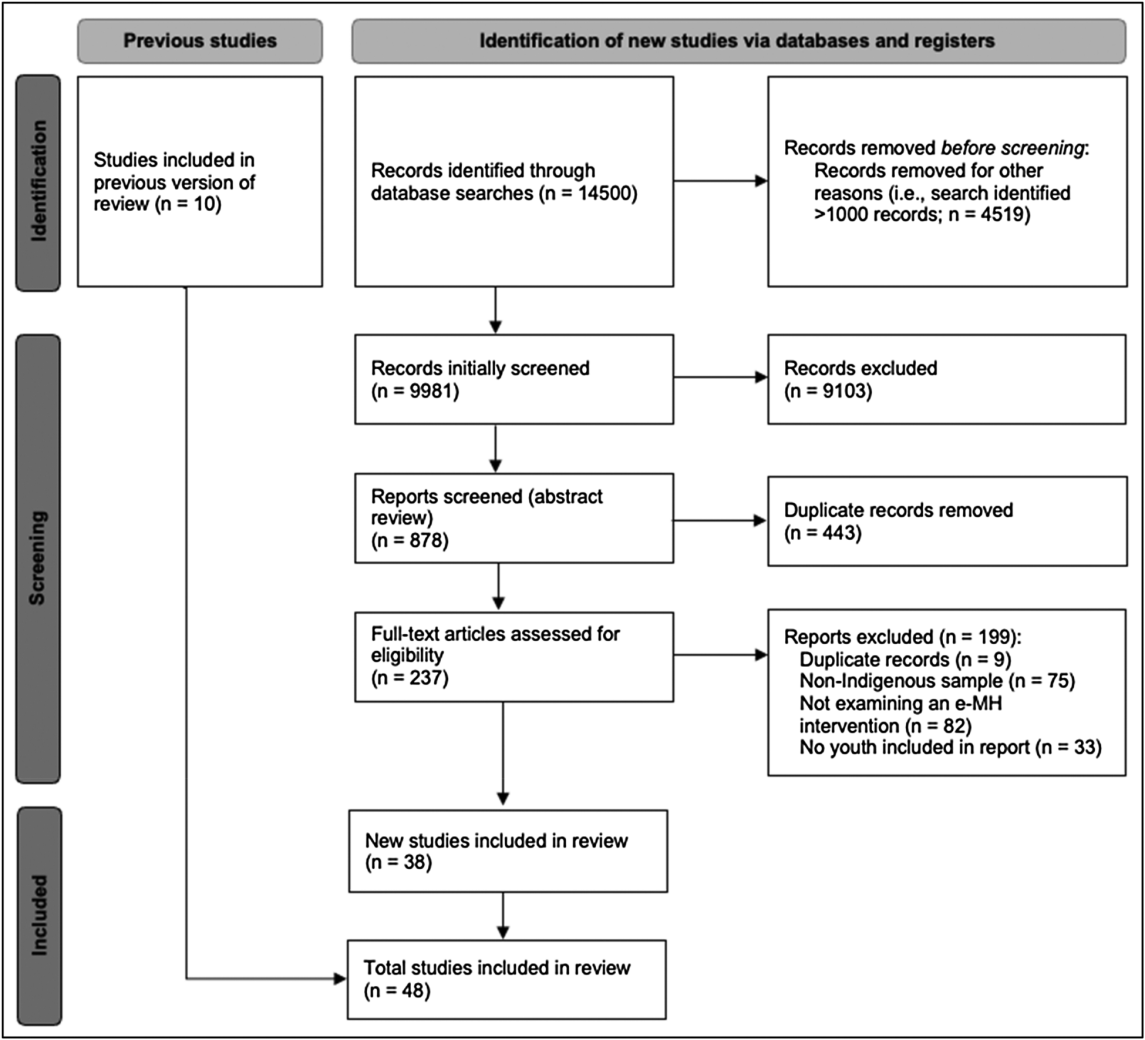
Literature review process via the preferred reporting items for systematic review and meta-analyses (PRISMA) diagram.

A preliminary review of literature from 19 databases was completed by one reviewer. This review included grey literature and relevant reference lists. Building upon our original search string,^37^ we conducted this preliminary review of the literature to identify any new terminology that would support our search aims. Our original search string included 24 terms but was expanded to include additional terms from Povey et al.^38^ recently published systematic review protocol, which will examine the various methods used to involve Indigenous youth in the development and evaluation processes of e-MH interventions.^38^ These original and additional search terms can be found at the bottom of Table 1. Following this preliminary review, a systematic review of these databases was completed by one author from 29 April 2021 to 25 June 2021, beginning with an initial title and abstract screening, and followed by a full-text review. Table 1 also includes the databases and grey literature sources examined. Figure 1 describes the number of studies included in the final review, in accordance with the most recent Preferred Reporting Items for Systematic Reviews and Meta-Analyses (PRISMA) standards.^41^ Tables 2 and 3 include data related to population, intervention, comparisons, and outcomes. Finally, a two-eyed seeing framework was used to interpret the literature, which allowed for standards and criteria to be developed while considering both Indigenous methodologies, ways of knowing, and knowledge, in combination with Western science.^42^

**Table 1.** Databases and grey literature sources were reviewed.

Databases	Grey literature
Academic Search Premier APA PsycNET Cumulative Index to Nursing and Allied Health Literature (CINAHL) Cochrane Library Education Resources Information Center (ERIC) Evidence-Based Medicine (EBM) Reviews Indigenous Collection (Informit) Iportal: Indigenous Studies Portal JSTOR Nursing and Allied Health Premium PubMed ProQuest Dissertation and Thesis Database ProQuest One Literature ScienceDirect Scopus Social Services Abstracts Social Science Research Network University of Alberta Indigenous Academic Journals Web of Science	Aboriginal Healing Foundation Alberta Health Services App Library Assembly of First Nations Centre for Addiction and Mental Health (CAMH) Library Canadian Child Welfare Research Portal Canadian Coalition for the Rights of Children Centre for Children and Youth in Challenging Contexts CHEO Research Institute Child Welfare League of Canada ClinicalTrials.gov DANS EASY (Open Grey Repository) ementalhealth.ca First Nations Child and Family Caring Society Health Navigator New Zealand Institute of Mental Health Research (IMHR) Inuit Tapiriit Kanatam King's Western University Library McCreary Centre Society Mental Health Commission of Canada National Collaborating Centre on Aboriginal Health National Native Alcohol and Drug Abuse Program New York Academy of Medicine Grey Literature Report NHS App Library One Mind Psyberguide Ontario Centre for Excellence for Child and Youth Mental Health Ottawa Hospital Research Institute (OHRI) Pauktuutit – Inuit Women of Canada PLEA Community Services Practical Apps Royal Commission on Aboriginal People Scarborough Health Network (SHN) Mental Health App Library Thunderbird Partnership Foundation Youth Aboriginal Health Organization
Search Terms: ** Original search string:** ** **Population: *Aboriginal,* “First Nation*,” *Indigenous, Indian, Métis*, *Native*, and *Inuit.* ** **Intervention: *smartphone,* “*short message service*,” *SMS*, “*text message*,” and “*cellular.* ** **“*phone*,” “*cell phone*,” telemedic*, “*electronic health*,” *eHealth*, “*e-health*,” and “*mobile.*” ** **“*health*,” “*distance-based intervention*,” “*internet intervention*,” and “*internet-based*.” ** **“*intervention*,” “*telehealth*,” and “*teleintervention*.” ** Added search terms:** ** **Population: *Māori*, Pacific*, Hawai*, and *Alaskan* ** **Intervention: *mHealth*, “*m-health*,” “*digital mental health*,” and “*telemental health.*” ** **“*m-therapy*,” “*e-therapy*,” “*online therapy*,” “*computer-assisted therapy*,” and “*iCBT.*” ** **“*online self-help*,” “*web-based treatment*,” “web-based therap*,” and “*website*” ** ***intervention*.”	

**Table 2.** Summary of retrieved articles – directly relevant for youth.

Article	Population and study location	Study design	Description of eHealth intervention	Study outcomes
Bartgis and Albright^43^	86 American Indian/Alaskan Native participants (41 faculty/staff, 19 students, 10 high school educators, 16 middle school educators) from 19 geographically dispersed states within the United States	Surveys collected pre-training, post-training, and at a three-month follow-up to examine the usefulness of Kognito gatekeeper training simulations Quantitative outcomes reported	Kognito gatekeeper simulations (KGSs) are online role-play training simulations that allow trainees to explore different behaviors and outcomes with computer-driven avatars or virtual humans Simulations include mini-conversation games in which trainees interact with intelligent, fully animated, and emotionally responsive avatars experiencing psychological distress KGS is designed to teach learners four core skills of Motivational Interviewing: asking open-ended questions, providing affirmation, reflective listening, and summarizing clients’ self-assessments	Significant increase in participants’ preparedness, likelihood, and self-efficacy to help students in psychological distress at the post-training and 3-month follow-up in contrast to the pre-training scores Significant increase in the mean number of actual gatekeeper behaviors reported at the three-month follow-up compared with the pre-training reports The training was thought to be useful, well-constructed, easy to use, likely to help, and relevant
Bowen et al.^44^ (*methods for developing and adapting SmokingZine for use in this study published in Youth Voices Research Group, 2012*)	113 American Indian youth aged 12–18 attending a Native summer enrichment program in South Dakota, USA to prepare students for success in college	Randomized control trial: participants in the intervention group were given access to the SmokingZine website for 1 h per day for the 6-week summer enrichment program whereas participants in the control group did not have access to the website Quantitative outcomes were collected both immediately after randomization and 1 month later; factor analysis was also conducted	SmokingZine is a web-based smoking cessation/prevention intervention Based on their current smoking status, youth are guided to educational modules on either smoking prevention or cessation Modules help reinforce the values of non-smokers, enhance motivation and commitment to change, and help youth identify goals, barriers, and strategies for behavior change Modules include culturally relevant contexts and images	52% of intervention youth signed into the website at least once and overall, intervention users rated the site positively on the survey Proportions of youth in each smoking category did not differ from baseline to follow-up Among non-smokers, youth from the intervention group were more likely to report helping others quit smoking and were less likely to report intentions to try a cigarette than those in the control group Youth from the intervention group also had less positive attitudes about the drug effects of smoking than the control group
Bramley et al.^45^ (*this paper reports Māori specific analyses; results from all participants can be found in Rodgers* et al.*, 2005*)	355 Māori participants aged 19–33 (*M *= 25) from New Zealand; participants were currently smoking daily, interested in quitting, and able to send and receive text messages	Single-blind randomized control trial: participants in the intervention group received smoking-related information whereas the control group did not Quantitative outcomes reported	STOp Smoking by Mobile Phone (STOMP) service provides regular, personalized text messages with smoking cessation advice, support, and distractions A quit day was negotiated for each participant; participants received text messages on the following schedule: 5 per day were sent in the week leading up to the Quit day, as well as during the following 4 weeks; 3 per week were sent 6 weeks later until the end of the 26-week follow-up Māori text messages related to Māori language, support messages in Māori and English, and information on Māori traditions	Participants in the treatment group were more likely to have stopped smoking at 6 weeks than those in the control group Salivary cotinine testing showed no clear evidence for differing degrees of over-reporting of quit rates between the intervention and control groups Smoking cessation rates remained high at 26 weeks in the intervention group but also increased in the control group
Christie et al.^46^	Young people from Māori and Pacific communities, clinicians, and software designers in New Zealand were consulted for this study	eHealth platform design and development study First, an initial qualitative scoping process was used to collect feedback from youth from Māori and Pacific communities Next clinicians were consulted via interviews and focus groups and iterative development sessions occurred with a software design company Between these sessions were short sessions and in-depth workshops with mostly Māori and Pacific youth, which supported the incorporation of Māori values and cultural practices into the app Qualitative feedback used to design and develop an app	The Quest – Te Whitianga app was developed to support learning of CBT skills The Quest – Te Whitianga app consists of several components: a narrative story, a gratitude journal, relaxation and mindfulness activities, behavioral activation activities, and activities designed to support the development of problem-solving and interpersonal skills Researchers also incorporated an aesthetic to appeal to Māori and Pacific young people (i.e. Māori names and cultural significance of “guardians” such as indigenous birds and sea creatures, as well as Māori proverbs, were incorporated into the app's narrative story) To encourage daily use, the app uses gamification and a fantasy story line	App developed The next steps are to conduct a randomized controlled trial of the app to assess efficacy, acceptability, and engagement, however, results from this RCT are not yet available
Cwik et al.^47^	600 tribal members aged 18–34 from the White Mountain Apache and Navajo Nation communities in Arizona and New Mexico, USA; elder participants aged 65 or older will also be included in this study; participants must have self-reported alcohol or drug use in the past 6 months	Protocol for a randomized controlled trial with a 2 × 2 factorial design: experimental intervention groups will receive basic support services (e.g. referrals to medical care) plus additional supports, whereas the control group will receive only the basic support services Experimental intervention groups are: (1) a culturally appropriate motivational interviewing group, (2) a text-based COVID-19 symptom monitoring system group, and (3) a group receiving both culturally appropriate motivational interviewing and text-based COVID-19 symptom monitoring Quantitative outcomes reported	Participants in the two groups with access to the COVID-19 symptom text-based monitoring system will receive alerts, ways for participants to request home testing after the first symptoms, and Geographic Information System routing for those responding to alerts	The study is currently ongoing Primary outcome measures include the proportion of participants tested for COVID throughout the study, the time to test from symptom onset, changes in protective behaviors (e.g. social distancing), the number of days quarantined per month, and vaccine acceptance Secondary outcome measures include cultural identity and connectedness, substance use, depression, and anxiety
Firestone et al.^48^ (*main results published in Mhurchu* et al.,^49^ *included in* Table 3*; this paper examines additional quantitative factors of wellbeing with demographic variables*)	794 Pasifika adults; results presented for age group quartiles—groups of interest for this review were ages 18–24 years and ages 25–34 years	Two-arm, community-based, cluster randomized control trial: clusters of Māori and Pasifika were (separately) randomly assigned to the intervention group who had access to a mHealth program via smartphone app and websites, or to a control group who had access to a control version of the app that only collected baseline and outcome data Quantitative measures were collected through the app via baseline, 4-week, and 12-week questionnaires	The OL@-OR@ project is a culturally tailored mHealth program, co-designed between New Zealand health researchers and Māori and Pasifika communities The smartphone app was designed to support better health and wellbeing via nutrition, health behaviors, and increased awareness of community-level activities, resources, and social cohesion	At 12 weeks after baseline, the intervention group reported significantly higher “family and community” wellbeing than the control group; however, this finding was across all age groups (ages 18–45+) and was not specific to our age groups of interest Being of young age (25–34 years) sustained significantly improved relationships with the spiritual well-being factor, but not with any other well-being factors
Fletcher et al.^50^	20 young Aboriginal fathers aged 18–25 from one regional and two rural Aboriginal communities in New South Wales, Australia; all fathers had at least one young child at the time of enrollment	Feasibility study using a participatory design approach Aspects of the study included: discussions evaluating a range of websites that could be included in the webpage; filming the young fathers’ stories; testing text messages and mood tracking messages; examining community feedback and evaluation; and website dissemination Qualitative outcomes reported	Stayin’ on Track SMS4dads text messages were sent out on topics including the father-infant bond, fathers’ support of their partner and co-parenting, and fathers’ self-care 25 messages were sent out over a period of six weeks and five contained links for further support Mood Tracker text messages were also sent weekly, which allowed a mentor to contact participants to offer support and direct them to other services when moods were low	The most popular link included in the text messages (*Routines: Aboriginal and Torres Strait Islander parents*) received more than half of all clicks for further resources 90% of the fathers responded to at least one of the mood-tracking text messages The consensus from the community feedback and evaluation was overwhelmingly positive, and the project offers preliminary support for the feasibility of providing support to young Aboriginal fathers through mobile-phone-based text-messaging and mood-tracking programs
Hanson et al.^51^	15 key informants aged 27–78 who were either American Indian (n = 14) or African American (n = 1) reviewed existing intervention 15 American Indian/Alaskan Native teens aged 15–19 examined the acceptability and suggested modifications for the final product Participants in this study were living in an American Indian/Alaska Native community in the Great Plains, USA	Two-tiered, qualitative process used to adapt existing intervention Key informant interviews were conducted to review an existing intervention; after modifications were made, focus groups were conducted to ensure validity and to finalize the intervention Qualitative outcomes reported	The CHAT program is an alcohol-exposed pregnancy (AEP) prevention program The CHAT program was adapted from the CHOICES program, which uses several motivational-interviewing focused activities (i.e. readiness rulers, decisional balance exercises, temptation/confidence exercises, and goal setting) to reduce AEP risk The CHAT program is hosted on a secure Web application that is interactive and user-guided	Focus group participants believed the CHAT program was acceptable, necessary, and feasible for teens in their community Focus group participants also felt that providing the program via the Internet might be more acceptable due to the sensitive nature of the topics Focus group participants found the information on birth control and alcohol use was new and informative Focus group participants were also very accepting of an optional visit to a healthcare provide to discuss topics in the program in further detail Focus group participants responded positively to the online chat intervention
Howson et al.^52^	Six mothers of teens aged 13–17 in New Zealand; three mothers identified as Māori, one as Pasifika, and the other two as Pakeha/New Zealand European	User experience co-inquiry (a form of think-aloud inquiry) via clinician-led, semi-structured interviews Qualitative descriptive outcomes reported	The harmonized smartphone app was co-developed with taitamariki (young people) to be Māori centered and inclusive Harmonized app is an mHealth intervention in the form of a private social network co-developed to promote healthy and safe partner relationships for 13–17 years old The harmonized app involves whanau (family) as safe people from whom taitamariki (young people) can seek advice and support from	The three themes identified were usefulness (e.g. access to a safe person), usability (e.g. moderation or monitoring of posts), and whanau context (e.g. Māori cultural perspective)
Jongbloed et al.^53^	200 Indigenous people aged 14–30 living in Vancouver and Prince George, British Columbia, Canada; participants included if they self-identified as smoking or injecting drugs such as crystal methamphetamine, opiates, crack or cocaine	Study protocol for a two-site, two-arm, parallel-group Zelen pre-randomized controlled trial: participants in the intervention group will receive the mHealth intervention whereas participants in the control group will receive standard care Participants will complete baseline assessments as well as follow-ups at 6 and 12 months	Cedar Project WelTel mHealth study uses two-way supportive text messages delivered in a community-based setting to reduce HIV vulnerability Intervention consists of a support package that includes: a mobile phone and cellular plan, weekly two-way text messaging, and support from Cedar Case Managers who follow a culturally safe approach that acknowledges both trauma and strengths Each week a check-in text message is sent, which is followed up with by the Cedar Case Managers that differ depending on the participant's type and timing of response	The study is currently ongoing The primary outcome measure will be HIV propensity scores at 6 months Secondary outcomes will be HIV propensity at 1 year, HIV risk, resilience, psychological distress, access to drug-related services, and connection to culture measured both at 6 months and 1 year
Jongbloed et al.^54^	131 Indigenous people aged 14–30 living in inner-city settings in British Columbia, Canada; participants included if they have used drugs	Mixed-methods exploratory study Quantitative comparative statistics and qualitative rapid thematic analysis on responses collected during the enrollment period of a larger study	Cedar Project WelTel mHealth was designed to support access to health care for Indigenous people who have used drugs while living with, or being vulnerable to HIV This program provided young Indigenous people who have used drugs with a mobile phone and a phone plan to connect them with case managers Also included were weekly two-way text messaging and support from case managers	93.2% of participants believed using a mobile phone for health would be invaluable, and mHealth acceptance was similar between those who owned a phone and those who didn’t Participants anticipated a diverse set of benefits from mHealth (e.g. connection for support; access to healthcare and emergency services)
Katapally^55^	76 Indigenous youth citizen scientists aged 13–18 from two rural First Nations reserves in Saskatchewan, Canada	Protocol and initial findings for a mixed-methods five-year longitudinal active living community trial titled the “Smart Indigenous Youth Initiative” Study pilot conducted in 2019 Baseline focus groups were conducted followed by a four-month intervention period Smartphone apps used in tandem with land-based active living programs during the intervention period	Custom-built SMART Indigenous Youth smartphone app aims to promote mental health, minimize substance abuse, and prevent suicide among Indigenous youth At baseline, the app was used to collect a combination of traditional validated measures and ecological momentary assessments During the intervention, the app was used to capture the perception and impact of land-based activities through time and user-triggered ecological momentary assessments	The study is currently ongoing Quantitative data captured physical activity, sedentary behavior, mental health, and substance abuse among other behaviors and outcomes Initial findings depict the overarching importance of culture, identity, history, and language, where land-based activities such as canoeing provided youth with a sense of purpose and identity, thus playing a role in improving youth mental health Data collection for the full five-year community trial will be published once available
Kerr et al.^56^	41 participants included teachers, coaches, counselors, and tribal health personnel with varying levels of experience responding to someone with suicidal ideation; 35 participants completed the pre- and post-surveys; 22 participants went on to complete the six-month follow-up survey Participants lived in various regions of the USA	Blocked randomized pilot study with two study arms: participants in the control study arm completed webinar/video training, and reviewed handouts; participants in the intervention arm did the same, and also participated in interactive role-play with a coach that took place via text message two weeks after the training Surveys were collected both immediately after the intervention and at a six-month follow-up Both quantitative surveys and qualitative role-play transcripts were reported	Web-based training intervention (*Responding to Concerning Posts on Social Media*) for adults who work with Native youth Intervention included: a self-paced webinar with supporting materials, a video introducing a response plan, summary handouts, and a coached role-play scenario	Participants viewed the training intervention positively and 100% indicated they would recommend it to their peers Even after six months, significant improvements were reported across several measures of training efficacy (i.e. contacting youth who post-concerning messages, confidence in starting conversations with youth, confidence intervening when a youth witnesses a concerning post, and confidence in referring youth to a mental health provider) Participants reported an increased number of clinical referrals at six months post-training Analysis of the coached role-play revealed three communication styles (collaborative, directive, and non-inclusive) when responding to youth who had viewed concerning posts)
Kypri et al.^57^	AQ: Yeaer 1789 Māori students aged 17–24 from seven of New Zealand's eight universities	Double-blind, multi-site, randomized controlled trial: participants in the intervention group received web-based personalized feedback, while participants in the control group completed only basic screening Parallel designs were conducted with Māori and non-Māori students Quantitative outcomes were reported	The web-based intervention provides personalized feedback on alcohol consumption scores Further web pages were provided that offered facts about alcohol, tips for reducing the risk of alcohol-related harm, and where further medical help and counseling support can be found Intervention was developed iteratively with consultation from both Māori and non-Māori students	Significant decreases were observed in the frequency of drinking, volume of alcohol consumed, and academic problems for the intervention group The intervention group also had a much lower prevalence of exceeding recommended limits for chronic harm (but not acute harm) relative to controls The intervention group was also 35% less likely to exceed the recommended weekly consumption limits
Poole et al.^58^	698 American Indian/Alaskan Native aged 18–34 living in the USA with mild, moderate, or severe risk of suicidality	Protocol for a randomized interventional clinical trial: participants in the placebo comparator group will receive the usual care prescribed in the Screening, Brief Intervention, and Referral to Treatment (SBIRT) model; participants in the experimental group will receive the usual SBIRT care as well as caring text messages (SBIRT +12) for a 12-month period following identification of suicide risk Quantitative outcome measures will be collected at baseline, 6, and 12 months	The SBIRT model consists of existing mobile phone technologies previously shown to promote resilience and incorporate the protective benefits of social connection The SBIRT +12 model consists of the SBIRT model as well as caring text messages adapted from empirically based, effective interventions for suicide prevention among American Indian/Alaska Native young adults	The study is currently ongoing Primary outcome measures will be changes in suicidal ideation, changes in self-reported suicide attempts, and changes in hospitalizations and behavioral health treatment Secondary outcome measures will be changes in social connectedness, as well as SBIRT retention and uptake of referral to therapy
Pacheco et al.^59^	41 American Indian tribal college students aged 18 or older (*M* = 29.3 years) in the USA All participants identified as smokers	Focus groups were conducted to support program development for a web-based smoking cessation program specifically for tribal college students Qualitative outcomes (i.e. thematic statements) were reported and used in the final determination of wording during the development of an online version of a pre-existing in-person smoking cessation program	Internet All Nation Breath of Life (I-ANBL) was developed to provide a web-based version of the pre-existing culturally tailored All Nation Breath of Life (ANBL) program, which supports smoking cessation in-person Focus group feedback guided the development of the website and audio-visual content The online platform selected for the program was one that students were already familiar with from their classes at the tribal college	Subthemes regarding program development from the female focus groups included: incorporating the use of visuals, videos, interactions, and incentives, and incorporating familiar and consistent support from peers and facilitators Subthemes regarding program development from the male focus groups included: incorporating a variety of visuals and cultural designs in bright/bold colors; incorporating money, food, and nicotine replacement therapy aids as incentives; including program content that discusses recreational versus traditional/ceremonial use; and connecting participants with facilitators who are former smokers
Povey et al.^38^	45 Aboriginal and Torres Strait Islander youth aged 10–18 from three sites in the Northern Territory of Australia	The participatory design research approach included a series of co-design workshops to analyze, synthesize, and integrate perspectives and preferences of e-mental health tools in an iterative review process Qualitative and quantitative outcomes were used to draft a new culturally responsive e-mental health resource in collaboration with Aboriginal and Torres Strait Islander youth	E-mental health tools reviewed by youth in the co-design sessions included: the Stay Strong App, the iBobbly App, the Yarn Safe Website, the WICKD Assessment App, Proppa Deadly podcasts, Italk health promotion cartoons, the TRAKZ Flipchart, and the AIMhi Yarning about Mental Health video	Young people were technologically competent and were using the internet to source health information Participants viewed apps as a way to anonymously seek help, normalize their experiences, and combat feelings of loneliness Participants thought e-mental health tools might be less suitable for those with severe mental illness Participants identified several features that engaged them (e.g. stories about positive change), sustained their interest (e.g. options for customization and personalization), and facilitated their access, help-seeking, and safety (e.g. security features)
Ritvo et al.^60^	168 youth aged 18–30 50% of the sample will be First Nation from Canada, and all will have been diagnosed with major depressive disorder	Protocol for a randomized controlled trial: participants in the experimental group will receive standard psychiatric care in combination with an online therapy program, while participants in the control group will receive standard psychiatric care alone Quantitative measures will be examined	The CBT-M software program is accessed online The program includes online workbooks covering multiple topics (e.g. Living By Your Truths, Loss and Grief), as well as navigation-coaching delivered through phone and text-message interactions Participants are also given a Fitbit-HR Charge 2 that assesses physical activity and heart rate The program uses CBT mindfulness contents that address specific symptoms and generic depressive experiences	The study is currently ongoing The primary outcome measure is the Beck Depression Inventory Secondary outcomes assess anxiety (Beck Anxiety Inventory), depression (Quick Inventory of Depressive Symptomatology; 24-item Hamilton Depression Rating Scale), mindfulness (5-Facet Mindfulness Questionnaire), and pain (Brief Pain Inventory)
Rushing and Gardner^61^	30 American Indian/Alaska Native youth from the USA aged 15–24 consulted prior to intervention adaptation 32 American Indian/Alaska Native youth aged 16–29 tested the intervention 14 American Indian/Alaska Native youth provided feedback on the intervention drafts Adult topical experts and staff at Indigenous youth-serving organizations also included in the process 67 AI/AN youth, parents, and tribal health educators completed surveys after watching the final video intervention	Formative research activities were carried out using focus groups and key informant interviews following the ADAPT-ITT Model (Assessment, Decision, Adaptation, Production, Topical Experts, Integration, Training, and Testing) Needs assessment, reviews, and testing were conducted before collecting survey responses on the final video-based intervention Both qualitative and quantitative outcomes reported	Video-based HIV/STI intervention designed and culturally adapted from two evidence-based interventions for HIV prevention to support heterosexual and LGBTQ-2S American Indian/Alaska Native teens and young adults Based on participants’ feedback, a culturally tailored intervention toolkit was produced The toolkit included: a User's guide, the Native VOICES video, condom and dental dam demonstration videos, and a selection of condoms and dental dams	Youth from focus groups and interviews recommended several changes to improve age and cultural appropriateness, as well as LGBTQ-2S inclusivity Survey responses from those who watched the final intervention indicated that most perceived the video to contain culturally appropriate information for AI people and trustworthy information Respondents also indicated the intervention portrayed topics that would work for them in real-life situations with relatable characters 73% of respondents were more likely to get tested for HIV/STIs and 66% were more likely to use condoms after watching the video Future work will include a randomized control trial to assess the efficacy of the intervention
Rushing et al.^62^	In Phase 1, 14 American Indian/Alaskan Native men aged 19–24 living across 13 states in the USA were included In Phase 2, 16 AI/AN young men with a history of alcohol use and violence, as well as eight topical experts, were included In Phase 3, 1000 AI/AN teens and young adults aged 15–24 from across the USA are being recruited	The five-year community-based participatory research project that occurred in three phases In Phase 1, qualitative interviews were conducted to obtain relevant information for designing a messaging campaign addressing violence and alcohol misuse In Phase 2, qualitative surveys were used to provide feedback on the tone, content, and frequency of planned text messages and video episodes In Phase 3, a randomized controlled study is currently being conducted: youth in the intervention group receive 8 weeks of BRAVE text messages and youth in the control group receive 8 weeks of Science Technology Engineering and Math (STEM) text messages	The BRAVE intervention includes a text message sequence and role model videos designed to address violence and substance misuse, improve help-seeking skills, and promote cultural pride and resilience for American Indian/Alaska Native young adults A Native-owned film crew, Sky Bear Media, worked iteratively with feedback from youth and topical experts to develop, test, and revise the script for the videos	In Phase 1, participants identified drug and alcohol use as contributors to violent behaviors, expanding the scope of the messages to include drug and alcohol users In Phase 2, American Indian/Alaska Native participants thought the intervention was relatable and helpful, while topical experts suggested emphasizing the influence of family histories and encouraging youth to reach out to trusted mentors Results from Phase 3 will be made available once the RCT is complete
Serlachius et al.^63^	In Phase 1, 20 young people aged 16–30 from New Zealand will be recruited, and at least 10 will be Māori or Pacific young people In Phase 2, 90 young people from New Zealand aged 16–30 years will be recruited, and at least 40% (i.e. 36) will be Māori or Pacific young people	Protocol for a two-phase mixed methods study aiming to evaluate the acceptability and effectiveness of The Whitu: 7 Ways in 7 Days app In Phase 1, 20 young people (10 Māori or Pacific) will provide qualitative feedback in focus groups In Phase 2, 90 young people will participate in a randomized waitlist-controlled trial with questionnaires at baseline, 4 weeks, and 3 months	The Whitu: 7 Ways in 7 Days well-being app was designed to promote coping skill development during and immediately following the COVID-19 pandemic Components of the app are to be completed over the course of one week Users receive daily push notifications reminding them to complete one module per day and to practice preferred exercises from previous modules The app is based on CBT, psychoeducational, and positive psychology practices, allowing users to choose the strategies that work best for them individually Cross-platform app developed to function with both Android and iOS operating systems	The study is currently ongoing Primary outcomes include quantitative changes in emotional well-being (WHO-5 scale) and mental well-being (Short Warwick-Edinburgh Mental Well-Being Scale) Secondary outcomes include quantitative changes in depression (Centre for Epidemiological Studies Depression Scale), anxiety (Generalized Anxiety Disorder seven-item scale), stress (Perceived Stress Scale), self-compassion (Self-Compassion Scale – Short Form), sleep (Sleep Quality Scale), and user engagement (App Subjective Quality and Perceived Impact subscales of the uMARS)
Shand et al.^64^ (*This study follows the pilot randomized controlled trial conducted by Tighe* et al.^65^)	289 Aboriginal or Torres Strait Islander participants aged 16 or older will be recruited from six regions (Broome, Darwin, Darling Downs, Hunter New England, La Perouse, and the Murrumbidgee region) in Australia	Protocol for a two-arm randomized controlled trial to test the effectiveness of the iBobbly app (version 2) in reducing levels of suicidal ideation immediately post-intervention: participants in the intervention group will receive access to the app while participants in the control group will be wait-listed This study involves an updated version of the iBobbly app (Tighe et al.^65^) following community consultation Quantitative measurements will occur at baseline, post-intervention (6 weeks after baseline), and a follow-up (6 months after baseline)	iBobbly is a self-help app developed to reduce suicidal ideation and plans iBobbly uses trans-diagnostic content from CBT (i.e. ACT, MBCT, and DBT) iBobbly was developed in consultation with Indigenous Australians and was designed to be an engaging and interactive method of delivering evidence-based therapy iBobbly was also designed to be accessible for people with lower literacy skills via the use of images, animations, and voice-overs, as well as minimal use of text Participants were provided with a tablet that had the app pre-downloaded; once downloaded, the app doesn’t require internet for ongoing use	The study is currently ongoing Outcomes will include changes in suicidality (Suicidal Ideation Attributes Scale), help-seeking (General Help-Seeking Questionnaire; Resource Use Questionnaire), depression symptoms (Patient Health Questionnaire), distress tolerance (Distress Tolerance Scale), interpersonal needs (Interpersonal Needs Questionnaire), and assessment of quality of life (Assessment of Quality of Life Scale)
Shrestha et al.^66^	700 American Indian or Alaskan Native women aged 16–20 who and living in urban areas of the USA and are not pregnant	Protocol for a randomized controlled trial comparing the effectiveness of a culturally adapted mobile health intervention to prevent alcohol-exposed pregnancies: participants in the intervention group will complete the Native WYSE CHOICES prevention program that has been translated for mHealth delivery, while participants in the control group will complete activities (e.g. quizzes, interactive games, and videos) designed under different topics than the intervention group	Native WYSE CHOICES is an alcohol-exposed pregnancy prevention program that translates a pre-existing evidence-based intervention targeting high-risk alcohol use during pregnancy into an mHealth intervention This intervention will include activities such as self-completed risk assessments when it comes to alcohol-exposed pregnancies and goal-setting	The study is currently ongoing Primary outcomes will include alcohol use and contraceptive use measured at baseline, 2 months later, 6 months later, and 12 months later
Stephens et al.^67^	1030 American Indian/Alaska Native teens and young adults aged 15–24 from the USA	Randomized controlled trial: participants in the intervention condition received 8 weeks of BRAVE text messages, while participants in the control condition received 8 weeks of STEM text messages After completing one arm of the study, participants completed the other arm with the next set of text messages	The BRAVE campaign incorporated three to five text messages per week, including one role model video per week and a related image This program was designed to amplify and reinforce healthy social norms and cultural values, teach suicide warning signs, prepare youth to have difficult conversations with peers and trusted adults, encourage youth to access mental health resources, destigmatize mental health services, and connect youth to trusted adults	Mental health-related outcomes from this study are reported for another paper, while this paper presented user analytics and overarching lessons learned This study had an 87% retention rate, and incentivization was proposed to contribute to this high level of retention Participant engagement and interaction with the study messages dropped off over time; authors propose incorporating a ‘pause’ or break in the program might be a promising strategy for future campaigns Text messages that included “a call to action” had greater response rates than those that did not
Taualii et al.^68^	In Phase 1, 12 Urban American Indian/Alaska Native youth aged 12–18 from various tribal affiliations in the USA were included In Phase 2, 13 American Indian/Alaska Native youth aged 12–18 were included	Two-phase pilot study Phase 1 involved focus groups to review and adapt an existing web-based program for use with American Indian/Alaska Native youth Phase 2 compared the adapted and original websites in another sample of American Indian/Alaska Native youth Qualitative outcomes reported	Web-based zine intervention tool adapted and modified from an existing web-based and youth-focused smoking cessation program to make it appropriate for young urban American Indian/Alaska Natives	In Phase 1, several ideas were provided by the focus groups to support website redesign that's tailored specifically to American Indian/Alaskan Native youth (e.g. changing all graphics to be specific to Native culture; adding or changing content to reflect Native customs; distinguishing between tobacco use for ceremonial and non-ceremonial use; and recognizing ceremonial uses of tobacco as positive and important to the heritage of Native people) In Phase 2, participants perceived the website components to be easy to use and understand, provided suggestions for specific improvements, and believed the site might help with tobacco prevention or cessation
Tighe et al.^65^	61 Indigenous Australians aged 18–35 (*M *= 26.25) from remote communities in Western Australia Participants included had suicidal thoughts	Pilot randomized controlled trial: participants in the intervention group received access to the iBobbly app, while participants in the control group were waitlisted Quantitative outcomes were collected at baseline, at the beginning of the intervention, and at a six-week follow-up (end of intervention)	iBobbly app delivered acceptance-based therapies including acceptance and commitment therapy Targeting suicidal ideation, depression, psychological distress, and impulsivity with three suicide self-assessments completed over six weeks Indigenous artwork and graphics are included to represent key messages of therapeutic content Audio developed to overcome low literacy and follow cultural protocol Developed in partnership with Indigenous community members from the Kimberly region in Western Australia	Changes in preintervention and postintervention suicidal ideation scores (Depressive Symptom Inventory – Suicidality Subscale) were significant for the iBobbly group but were not significantly different from the waitlist group Significant reductions in the depression scores (Patient Health Questionnaire 9) and psychological distress scores (Kessler Psychological Distress Scale) for the iBobbly group when compared to the waitlist group No differences were observed in impulsivity (Barratt Impulsivity Scale) Waitlist participants improved after 6 weeks of app use
Tighe et al.^69^	13 participants aged 19–29 (*M *= 24.15) who were part of the larger cohort in the Tighe et al.^65^) study were interviewed here Data from 40 participants from the Tighe et al.^65^) study also examined	Qualitative interviews were conducted after the Tighe et al.^65^) trial to determine whether the iBobbly app was deemed effective, culturally appropriate, and acceptable by participants Quantitative usage data from 40 participants was also analyzed and presented here	The same suicide prevention app discussed in the row above (iBobbly) The app was purely self-directed and relied solely on user motivation	Regression analyses from usage data demonstrated that participants with higher needs used the app more frequently Interview participants enjoyed the accessibility and privacy of the app but noted it might not be enough in the face of strong emotions Participants perceived the app to create a safe space for Indigenous people to explore mental health coping strategies without shame Participants felt the app reduced their distress and helped distract them from their thoughts
Vigil-Hayes et al.^70^	Researchers in the process of assembling a community advisory board including Navajo, Hopi, and other Southwestern tribal healthcare professionals and educators living in the USA who have experience working with Native American adolescents The pilot study will target Navajo students aged 14–19 living in Arizona, USA	A community-based participatory research approach to include Native American psychologists, community health workers, and educators as co-designers of the intervention activities and gaming mechanisms Currently working to establish a pilot to be conducted with youth	ARORA is a positive-psychology-focused behavioral intervention designed to promote engagement in targeted social and emotional learning activities ARORA is delivered over a networked, geosocial mobile game that incorporates both augmented reality technology and cultural themes Use of ARORA does not require a robust or high-performance connection to the internet Designed to be culturally relevant through the integration of words and phrases from the Navajo language, Navajo cultural values and images, and input from Navajo collaborators	The pilot study is currently in progress Primary outcomes will be measures of coping skills and interpersonal skills
Volpe et al.^71^	Frontline mental health workers working with young people experiencing mental health and behavioral issues from 14 different sites in three regions in Nunavut, Canada consulted a psychiatrist from Toronto, Canada	Interpretive interactionist framework used to collect qualitative information on personal experiences via participant observation, individual interviews, and focus groups	TeleLink Mental Health Program is designed to support Inuit youth with complex needs via professional-to-professional case consultation Teleconferencing enabled 12 psychiatric consultations and four 2-hour-long continuing education sessions	Videoconferencing technology was an effective way of providing psychiatric consultation services to remote communities Factors enhancing (e.g. advance scheduling) and hindering (e.g. communication) the program are discussed Recommendations are provided to facilitate gaining access, enhancing the participant experience, delivering continuing education, and ensuring stable and confidential technology
Yao et al.^72^	192 American Indian and Alaska Native youth aged 15–24 years old from the USA	An iterative, formative research process using a before-after study design was used to assess the impact of the text messages on condom use and STI/HIV testing behavior The study involved 97 SMS messages delivered over 9 months; these included 32 intervention messages 36 survey questions, and others Quantitative surveys were collected at baseline, 3, and 6 months	We R Native is a multimedia health resource for Native teens and young adults that includes: a website, a text messaging service, a YouTube channel, and social media pages This platform had an existing relationship with American Indian/Alaska Native teens The SMS intervention was designed using insights from the health belief model, social cognitive theory, and the theory of planned behavior Formative research during intervention development captured the sexual health needs of American Indian/Alaska Native youth of varying ages, genders, and sexual orientations while ensuring cultural relevance and sensitivity	Condom use attitude, condom use behavior, and STI/HIV testing intention improved after the intervention Frequent condom use increased from 30% to 42% and this was retained at least 3 months post-intervention Intentions to get tested for STI/HIV after changing sexual partners increased from 46% to 58% post-intervention

**Table 3.** Summary of retrieved articles—not specific to youth, but youth included in the study.

Article	Population and study location	Study design	Description of eHealth intervention	Study outcomes
Buchwald and McPherson^73^	346 American Indian participants aged 18 or older living in the states of Alaska, Oklahoma, Wisconsin, Minnesota, and New Mexico, of the USA Participants must be current daily smokers and be interested in quitting smoking within the next 30 days	Protocol for a randomized, single-blinded controlled trial: participants in the intervention group will participate in a text-message-based smoking cessation program for individuals who call state quitlines; participants in the control group will receive treatment as usual provided from the quitlines Participants will complete questionnaires at baseline, as well as 6, 12, 18, and 26 weeks after their target quit date Quantitative outcomes will be reported	The AI STOMP smoking mobile program is adapted for American Indians from the previously successful STOMP program used among Māori young adults in New Zealand Participants using AI STOMP will receive culturally tailored messages encouraging them to quit smoking Participants will receive texts on the following schedule: 4 messages per day for 1 week prior to the target quit date, 4 messages per day for 4 weeks following the target quit date, 3 messages per week for 20 following weeks	The study is currently ongoing The primary outcome measure is the number of participants who change smoking status as measured by self-report at 4-time points over 6 months
Campbell^74^	53 American Indian or Alaskan Native participants aged 18 or older with recent alcohol or drug use and within their first 30 days of current outpatient treatment episode Participants will be recruited from the USA	Protocol for a randomized controlled trial: participants in the intervention group will receive a culturally adapted therapeutic education system in addition to standard outpatient addiction treatment, while participants in the control group will receive the standard outpatient addiction treatment only Quantitative outcomes will be reported	The Therapeutic Education System – Native Version (TES-NAV) is a web-delivered psychosocial intervention for substance use disorders adapted for American Indians/Alaskan Natives The intervention is grounded in community reinforcement as well as contingency management	The study is currently ongoing The primary outcome measure will be the consecutive weeks of drug/alcohol abstinence across a 12-week period
Daley and Swimmer^75^	500 American Indians aged 18 or older from the USA who smoke at least 1 cigarette per day	Protocol for a randomized interventional trial: participants in the intervention group will receive a telephone-based version of the All Nations Breath of Life smoking cessation program, while participants in the control group will receive a non-culturally tailored comparison program Quantitative outcomes will be reported	Telephone All Nations Breath of Life is a culturally targeted smoking cessation program developed for American Indian communities The program includes individual telephone counseling, text messaging, and educational materials	The study is currently ongoing The primary outcome will be a 7-day point prevalence abstinence from smoking cigarettes at 6 months post-baseline Secondary outcomes will be continuous abstinence and reduction in smoking both at 6 and 12 months post-baseline
Dignan et al.^76^	254 American Indians aged between 18 and 80 years (with 63% under the age of 50 years) in South Dakota, USA	A randomized trial with a 2 × 2 × 2 × 2 incomplete factorial design with 15 possible treatment combinations Participants were assigned to one of the 15 groups that included combinations of four interventions (Nicotine Replacement Therapy or NRT; pre-cessation counseling; post-cessation counseling; mHealth text messages) provided at one of two available intensity levels (minimal or intense) The treatment period spanned from the initial visit to the 18-month follow-up visit Quantitative outcomes reported	Text messages provided in the mHealth intervention type were provided in either minimal or intense levels of smoking cessation support All text messages were categorized into (1) generation information/statistics, (2) general motivation, (3) strategies, and (4) traditional American Indian perspectives All text messages were also cross-listed by the “type of message” (e.g. crave, tips, slip, mood, motivation and quotes, and for the phase of quitting) There were 438 different text message options, of which, about half were American Indian specific	The percentage of participants who were abstinent was highest (35%) among three groups: (1) those who received a combination of intense NRT and minimal remaining interventions, (2) those who received a combination of minimal NRT and intense pre-cessation counseling and mHealth, and (3) those who received a combination of intense pre-cessation counseling and minimal remaining interventions Receiving NRT was associated with increased odds of having stopped smoking at the 18-month assessment 61% of the 108 participants that remained at the study quit date (visit 5) were abstinent 88% of the 16 participants who remained at the 18-month follow-up visit were abstinent
Dingwall et al.^77^	15 mental health service providers and an elder working with Aboriginal and Torres Strait Islander people in rural and remote areas in the Northern Territory, Australia	Participatory action framework using semi-structured interviews to translate the Australian Integrated Mental Health Initiative (AIMhi) Motivational Care Planning (MCP) intervention into an electronic format Qualitative outcomes reported	The AIMhi program was developed to promote access to mental health treatment for Aboriginal and Torres Strait Islander people via mental health promotion and educational tools based on cross-cultural understandings of mental health and illness AIMhi tools were translated into an electronic format (i.e. the AIMhi Stay Strong App) with the aim of developing a more accessible, visually appealing, low-intensity intervention suitable for health worker-supported delivery This intervention centers on peoples’ strengths, worries, and goals for change	Participants responded positively to questions related to the app's acceptability (e.g. perceived cultural relevance), ability to support the engagement process and develop the therapeutic relationship (e.g. breaking down barriers), and broad applicability (e.g. people and settings) Specific to youth, participants thought that the app might open up a conversation with young people who might have difficulties engaging in certain conversations, that the app might be most useful for young people who could adopt it with greater ease, and that the app might be particularly useful for earlier intervention for youth
Gorman et al.^78^	21 American Indian/Alaska Native women of childbearing age (18–45 years old; no average age reported) in a Southern California community in the USA	Semi-structured interviews were conducted to examine perceptions of each section of the Web-based program Interviews conducted within several focus group sessions and information used in further development of the Web-based program Qualitative outcomes reported	Pre-existing web-based Screening, Brief Intervention, and Referral for Treatment (SBIRT) program designed to prevent risky alcohol use among women of childbearing age Information from focus group sessions used to modify elements of the SBIRT program to be more relevant for American Indian/Alaska Native women	Five key areas were identified to facilitate making the intervention culturally appropriate, understandable, accessible, and relevant to American Indian/Alaska Native women of childbearing age These areas were: including more personal and relatable information; emphasizing confidentiality; incorporating family and community orientation; tailoring content to American Indian/Alaska Native community; and including more information about how women's alcohol use can negatively impact children's health
Koziol-McLain et al.^79^	111 Māori women aged 16–60 in New Zealand who had experienced intimate partner violence in the past 6 months	Two-arm parallel randomized controlled trial: women in the intervention group had access to a web-based safety decision aid for one year, while women in the control group had access to a standardized list of resources without individual feedback or a tailored action plan Assessments took place at baseline, 3, 6, and 12 months after completion Quantitative outcomes examined	Web-based safety decision aid (*isafe*) for women experiencing intimate partner violence in Aotearoa New Zealand culture adapted from web-based decision aid used previously in the United States The website consists of three components: the first was a safety priority setting activity in which women could examine which priorities were most important to them; the second component was a danger assessment which provided immediate scored feedback on their level of danger; the third component was an interactive process using several local, region, and national resources to help women develop an individually tailored action plan	Intervention estimates depression symptoms (Center for Epidemiologic Studies Depression Scale – Revised) and for severity of intimate partner violence (Severity of Violence Against Women Scale) at all time periods favored the intervention but were not statistically or clinically significant No study-related adverse events were reported No differential intervention effect for violence or depression symptoms based on whether women were caring for children or not Significant intervention effect for reducing violence for Māori women at 6 months and 12 months Significant intervention effect for reducing depression symptoms for Māori women at 3 months but not at 6 months or 12 months
Mhurchu et al.^49^ (*see Te Morenga* et al.*, 2018 for the approach used in co-designing the mHealth tool and Verbiest* et al.*, 2018 for the published study protocol*)	1451 participants aged 18 or older (657 Māori and 794 Pasifika) from communities in New Zealand	Two-arm, community-based, cluster randomized control trial: clusters of Māori and Pasifika were (separately) randomly assigned to either the intervention group who had access to a mHealth program via smartphone app and websites, or to a control group who had access to a control version of the app that only collected baseline and outcome data Measures collected through the app via baseline, 4-week, and 12-week questionnaires Quantitative outcomes reported	The OL@-OR@ mobile health program (smartphone app and website) was co-designed with Māori and Pasifika communities in New Zealand to support healthy lifestyle behaviors The program provided information on healthy eating and physical activity, culturally relevant information, and links to local activities and services The program also supported users in setting goals to change their health behaviors and to identify the steps needed to reach those goals Regular motivational messages were also sent to encouragement	Relative to baseline, adherence to health-related behavior guidelines increased at 12 weeks in both the intervention and control groups However, the proportion of participants adhering to guidelines for physical activity, smoking, alcohol consumption, and fruit and vegetable intake did not differ between groups Exploratory analysis comparing intervention group participants who engaged with the program to the control group found that those in the former group showed significantly greater adherence to health-related behavior guidelines at 12 weeks than the control group
Montag et al.^80^	American Indian/Alaska Native women aged 18 to 45 (*M* = 28.6, *SE* = 0.5) who were of childbearing potential in the USA	Randomized controlled study: participants in the intervention group completed a web-based Screening, Brief Intervention, and Referral to Treatment (SBIRT) intervention while participants in the control group received treatment as usual (i.e. access to educational brochures about health) Assessments occurred at baseline, and at 1-, 3-, and 6-month follow-ups Quantitative outcomes were reported	eCHECKUP TO GO is a web-based brief assessment and intervention tool that aims to reduce risky drinking and vulnerability to alcohol-exposed pregnancies This intervention was tailored to American Indian/Alaska Native women in previous research After answering questions, participants received individualized web-based feedback at the end of the session relating to their alcohol-exposed pregnancy risk and other aspects of their drinking Participants were also provided with resources for additional information	Across the three main variables (number of drinks consumed per week, number of binge episodes in past 2 weeks, and high risk for alcohol-exposed pregnancy), all outcomes significantly decreased across time points, but did not significantly differ between the control and intervention groups Baseline factors associated with decreased risk of alcohol-exposed pregnancy at follow-up included: the perception that other women in the peer group had consumed a greater number of drinks per week; having reported a greater number of binge episodes in the past 2 weeks; and depression/impaired functionality
Nelson and Comtois^81^	1200 American Indian/Alaskan Native people aged 18 or older from four Native communities in the USA Participants must also be suicidal or have a documented or self-reported suicide attempt within the past year	Protocol for a randomized controlled trial: participants in the intervention group will receive usual care plus caring text messages sent via the caring contacts program, while participants in the control group will receive usual care alone Outcome measures will be collected at 12 and 18 months Quantitative outcomes will be reported	Caring Contacts is a suicide prevention program that supplements standard care by promoting human connectedness via text messages expressing care, concern, and interest Text messages express care and support, and are sent on the following schedule: the next day following the initial meeting, 6 weekly, 9 biweekly, 7 monthly; one each on birthday, holiday, and seasonal The version of Caring Contacts used in this study was culturally adapted through a collaborative process with tribal partners	The study is currently ongoing Primary outcome measures include suicidal ideation (Suicidal Ideation Questionnaire), suicidal attempt and self-injury counts, and suicide-related hospitalizations The secondary outcome measure is the lack of perceived social connectedness (Interpersonal Needs Questionnaire – Thwarted Belongingness Subscale)
Orr et al.^82^	487 American Indian participants aged 18 or older (*M* = 41.9, *SD* = 11.7) and nine American Indian students aged 18 or older (*M* = 32.0, *SD* = 15.24) in the USA	Rationale, design, and methods for a single-blind, randomized trial: participants in the intervention group received culturally tailored text messages while participants in the control group received care as usual Baseline and 6-month follow-up measurements collected The study was originally meant to recruit only American Indian tribal college students, however, there were only nine students enrolled after the first year of recruitment so recruitment was expanded to include smokers already using state quitlines	Text messages from a smoking cessation intervention previously used with Māori participants were adapted via focus groups of tribal college students for cultural appropriateness with American Indian participants Participants received 200 text messages (140 in the first 4–6 weeks, 60 in the following weeks) Texts began one week prior to the participant's quit date, after which intervention texts followed	The most common reason for non-participation for the tribal college students was that they were not ready to quit The study is currently ongoing Primary outcomes will be self-reported continuous smoking absence measured via the Fagerström Test of Nicotine Dependence and the Hooked on Nicotine Checklist Participants will also be asked additional questions regarding smoking behavior (e.g. number of cigarettes smoked, number of attempts at quitting)
Peiris et al.^83^	49 Aboriginal and/or Torres Strait Islander people aged 16 or older (*M *= 42, *SD *= 14) from Australia	Pilot randomized controlled trial: participants in the intervention group received access to the Can’t Even Quit App, while participants in the control group received usual healthcare smoking cessation services Baseline quantitative measures were collected along with follow-up visits at 4 weeks and 6 months 15 qualitative interviews with participants in the intervention group were conducted to assist with the explanation of outcomes observed in the trial	Can’t Even Quit App designed to support smoking cessation Comprises of a personalized profile and quit plan, text and in-app motivational messages, and a challenge feature allowing users to “compete” with each other	Only two participants from the intervention arm reported abstinence at either the 4-week or 6-month interview, and nobody had continuous abstinence at both time points Generally, higher numbers of participants from the intervention arm reported the use of supportive cessation services, however, these differences were not statistically significant No harm or unintended consequences observed Statistics indicate a low to moderate level of app usage during the trial Several contextual factors (e.g. everyday use of mobile technology), mechanisms for quitting (e.g. supports to assist with quitting), and trial outcomes (e.g. app engagement) were discussed in the interviews
Povey et al.^84^	Nine Aboriginal and Torres Strait Islander community members (*M *= 33, *SD *= 17) in Darwin, Northern Territory, Australia	Focus groups were conducted using qualitative methods and a phenomenological approach to determine the acceptability of two culturally responsive e-mental health apps (the AIMhi Stay Strong app and the iBobbly app) designed for Aboriginal and Torres Strait Islander people Thematic analyses were conducted and qualitative outcomes reported	The Australian Integrated Mental Health Initiative (AIMhi) Stay Strong iPad app uses therapist-guided strengths-based brief intervention integrating motivational interviewing and low-intensity CBT techniques The iBobbly suicide prevention app is based on acceptance and commitment therapy and uses mindfulness and values-based action strategies; it is designed as a self-driven assessment tool and offers support for emotion management and goal-setting	Specific benefits identified were: the opportunity to reach larger audiences; the ability to provide immediate access to help; the ability for an individual to have greater independence; and the possibility of anonymity Factors found to influence the acceptability of e-mental health apps for Aboriginal and Torres Strait Islander people included characteristics of the person (e.g. historical factors), characteristics of the environment (e.g. community awareness), and characteristics of the apps (e.g. ease of use)
Raphiphatthana et al.^85^	57 service providers working with First Nations at the primary care level (e.g. nurses, support workers, and psychologists) who received training in Darwin, Alice Springs, and remote Northern Territory communities of Australia	A qualitative approach embedded in a contextualist paradigm is used to understand the factors that influence the uptake of electronic mental health approaches in primary care workers working with First Nations Participants were either self-selected or selected by their organizations to attend electronic mental health training Following the training, participants completed a follow-up semi-structured interview to provide their perceptions and experiences of using the electronic mental health resources Qualitative outcomes reported	The eMHPrac project is used to support electronic mental health uptake via a number of strategies including web-based campaigning and the provision of electronic mental health training The training aims to raise awareness of validated electronic mental health tools that are responsive to First Nations cultures	Results presented here are those relevant to our specific population of interest; full results can be reviewed in the article The majority of participants perceived electronic mental health to be suitable and applicable for young people, so those who worked with youth were more receptive to the innovation Having a good understanding of how resources would be effectively used in a real-life context (e.g. working with a young person whose vocabulary differs from adults) was perceived to be important Lack of technological support (i.e. IT infrastructure within communities) was perceived as a difficulty for young people
Reilly et al.^86^	288 Aboriginal and Torres Strait Islander people aged 16 and over from geographically remote, regional, and urban locations in Australia Participants will have used methamphetamine at least weekly in the past 3 months	Protocol for a randomized controlled trial: participants in the intervention group will complete the online We Can Do This program whereas participants in the control group will be wait-listed while undergoing the same assessments as the intervention group until the 3-month control period is complete Baseline data will be collected for both groups, with follow-up data being collected on day 30, day 60, and day 90	The We Can Do This online web-based intervention consists of nine modules (e.g. Cravings & Triggers; Slip-ups) based on CBT and motivational interviewing treatment approaches, as well as advice from clinical and cultural advisors who guided the development towards ACT and narrative approaches Feedback from a clinical advisory group, a cultural advisory group, and a lived experience group was used to improve the intervention's acceptability and usability	The study is currently ongoing The primary outcome will be the number of days the participant used methamphetamine during the treatment phase, assessed using questions from the Australian Treatment Outcome Profile Secondary outcomes will be help-seeking (General Help-Seeking Questionnaire) as measured by the rate of referral and frequency, type, and duration of health service contact resulting from the use of the online intervention Other secondary outcomes will be readiness to change (Readiness to Change Questionnaire), psychological distress (Kessler 10), poly-drug use during the past month (Australian Treatment Outcome Profile), severity of dependence (Severity of Dependence Scale), days out of role, and usability and acceptability (Internet Intervention Adherence Questionnaire) of the online program
Sabri et al.^87^	Formative phase: 43 Native American survivors of intimate partner violence aged 18 or older from multiple tribes in the USA Randomized controlled trial: 500 Indigenous survivors of intimate partner violence aged 18 or older from diverse regions of the USA (½ urban dwelling and ½ living on tribal land)	The formative phase allowed for the original myPlan intervention to be culturally adapted Protocol for a randomized controlled trial described: participants in the intervention group will receive access to the culturally-tailored safety planning app while participants in the control group will receive usual safety planning resources via a control app Assessments collected at baseline, as well as 3-, 6-, and 12-months post-baseline Quantitative outcomes will be reported	Culturally adapted web-based safety decision aid/safety planning (ourCircle) intervention app designed to decrease the risk of future intimate partner violence and poor mental health, and increase empowerment The app provides education about healthy relationships and red flags for unsafe and abusive relationships The app also allows the user to complete danger assessment questions and provides immediate feedback on danger level The app then incorporates safety priorities into a tailored safety action plan with links to further resources and services The DA is culturally specific for each of the study target groups (i.e. immigrant, refugee, or indigenous)	The study is currently ongoing The primary outcome measure is change in severity and frequency of physical violence (Conflict Tactics Scale-2) Secondary outcome measures include changes in depression (Patient Health Questionnaire), symptoms of PTSD (Harvard Trauma Questionnaire), overall empowerment (Personal Progress Scale-Revised), and empowerment related to safety (Measure of Victim Empowerment Related to Safety)
Sinicrope et al.^88^	Stage 1A: 40 participants (30 Alaska Native people aged 19 or older who smoke and 10 stakeholders) Stage 1A, Phase 2: 40 new Alaska Native participants who smoke Stage 1A, Phase 3 Beta Test: 10 Alaska Native adult smokers Stage 1B: 60 participants All participants will be from the USA	Protocol for mixed methods study Stage 1A uses a mixed methods approach to develop the intervention through qualitative semi-structured interviews, quantitative surveys, and a beta-test Facebook group Stage 1B is a 2-arm, parallel-group, pilot randomized controlled trial: participants in the intervention group will receive access to the culturally relevant Facebook-delivered smoking cessation intervention, whereas participants in the control group will receive access to the state quitline program and treatment referrals only	Culturally relevant Facebook-delivered smoking cessation intervention that uses a digital storytelling approach adapted from a previously effective campaign (Centre for Disease Control Tips from Former Smokers) The intervention will be in a hidden and closed Facebook group A moderator (Alaska Native tobacco research counselor) will post once daily for 30 days, repeated over 3 months, with three to four daily check-ins to respond to comments from participants and encourage sharing of personal stories relevant to the quitting process and treatment engagement	The study is currently ongoing Primary outcome measures will include feasibility and biochemical verification of smoking abstinence at 1-, 3- and 6-month follow-ups Secondary outcome measures will include self-reported smoking cessation treatment utilization and abstinence from tobacco/nicotine products Culturally relevant mediators (i.e. relationship orientation and collaborative efforts in lifestyle change) will also be explored
Titov et al.^89^	780 Aboriginal and Torres Strait Islander users of MindSpot (*M *= 32.0 years) in Australia 49 Indigenous patients enrolled in the Wellbeing course 21 Indigenous patients enrolled in the Indigenous Wellbeing course	Prospective uncontrolled observational cohort study Quantitative characteristics compared between Indigenous users and non-Indigenous users of MindSpot Quantitative characteristics and treatment outcomes were compared between Indigenous users and non-Indigenous users of MindSpot	The MindSpot website was developed to improve the availability of mental health services for adults with anxiety and depression who are living in remote parts of Australia MindSpot provides free assessment and offers seven different treatment courses (the Wellbeing course, Wellbeing Plus for older adults, Mood Mechanic for younger adults, the Indigenous Wellbeing Course, courses for treatment of PTSD, OCD, and the disability and distress associated with chronic pain) The Indigenous Wellbeing course shares the same core content as the Wellbeing course but has been adapted to reflect how the experiences of Indigenous people can affect their mental health	Indigenous patients had higher symptom scores at assessment on each of the symptom measures including psychological distress (Kessler 10-item Scale), depression symptoms (Patient Health Questionnaire 9-Item), and symptoms of generalized anxiety, social phobia, panic, and PTSD (Generalized Anxiety Disorder Scale – 7-Item) at baseline There was a significant decrease from assessment to post-treatment on measures of psychological distress, depression symptoms, and symptoms of generalized anxiety, social phobia, panic, and PTSD There were no differences in treatment outcomes between Indigenous and non-Indigenous patients, regardless of the course that the Indigenous patients were enrolled in Treatment satisfaction measured at post-treatment was >97% for both groups

* *Note:* In the “Population and study location column,” *M* indicates age mean and *SD* indicates age standard deviation.

### Inclusion criteria

Articles were included in this review if they described any type of e-MH intervention designed to improve the mental wellbeing of Indigenous youth. Articles were also included in this review even if the participants included in the studies were not Indigenous youth, and this was to ensure that we captured e-MH interventions designed to improve the mental wellbeing of Indigenous youth *via their support networks* (e.g. family members, community members, healthcare workers, etc.), as seen in the FNMWCF^39^ and the ILCSDAH^40^ discussed above. Articles also had to be available in English and be published between 1 January 2000 and 1 June 2022. Youth was defined as being between 12 and 30 years of age. While Consolidated Standards of Reporting Trials (CONSORT) best practices indicate that only those sources that meet a particular threshold of quality should be evaluated in a systematic review, this was not completed as several studies would have been removed. This is in alignment with a framework by Short et al.^90^; all studies were included if they met the inclusion criteria.

## Results

### Study characteristics

**Figure 2.**
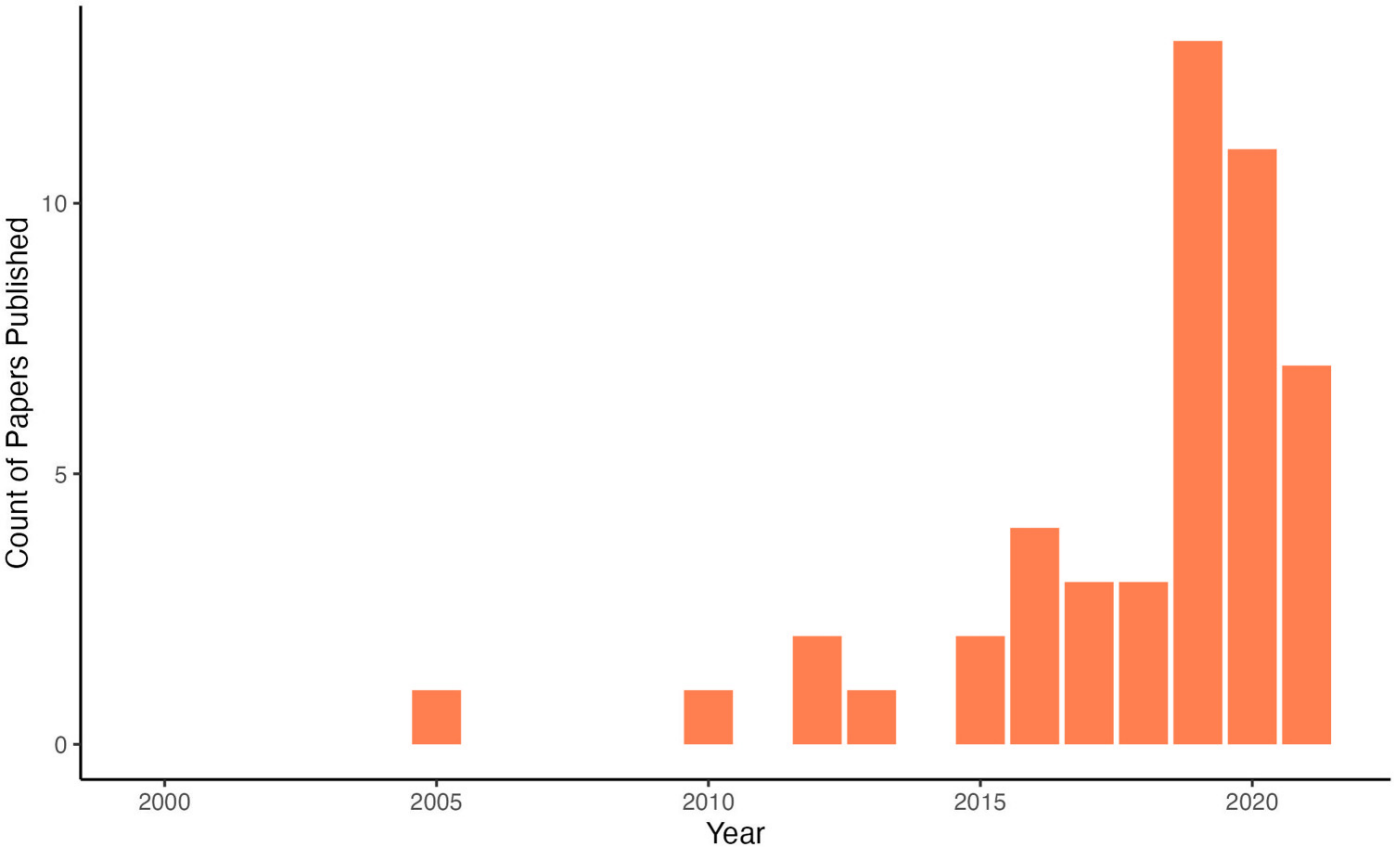
Count of papers published by year.

Forty-eight articles were included in this review, 38 of which were updated from the previous review.^37^ Figure 2 provides a visual of the increase in studies over the past few years. Table 2 describes 30 studies that aimed to support youth/young adults within the defined age range, while Table 3 describes 18 studies that included participants within the defined age range as well as participants older than the defined age range. Throughout each section in the results, studies from Table 2 will be referred to as “youth-specific studies” and studies from Table 3 will be referred to as “broader studies.”

#### Mental health concerns targeted

**Figure 3.**
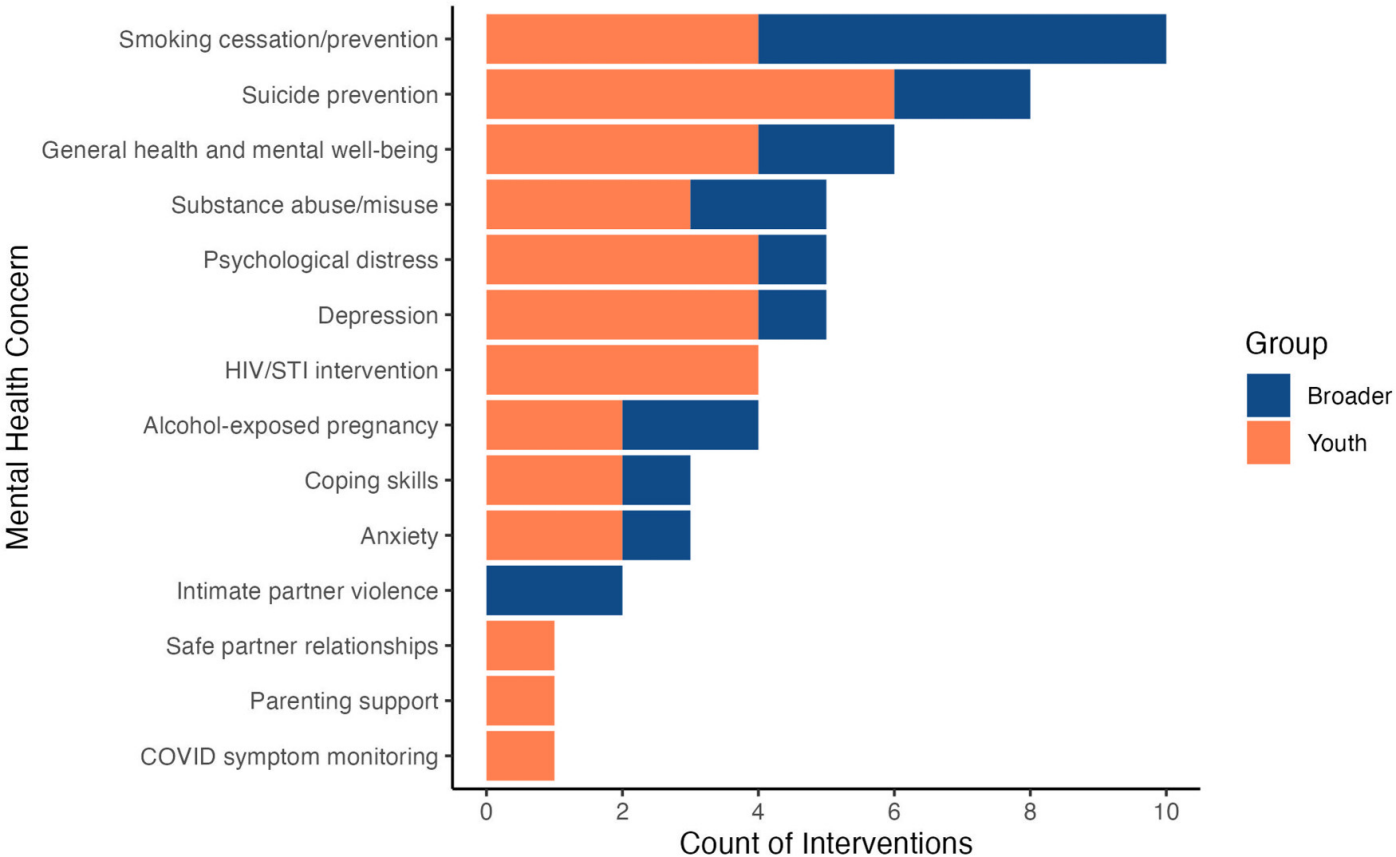
Count of interventions by mental health concerns.

Studies targeted a wide variety of mental health concerns. Several studies targeted more than one mental health outcome so study counts amount to more than the total number of studies initially reported (Figure 3). The mental health concerns examined in these interventions were: alcohol-exposed pregnancies,^51,66,78,80^ anxiety,^60,63,89^ coping skills,^46,70,84^ COVID-19 symptom monitoring,^47^ depression,^60,63,65,69,89^ general health and mental well-being,^48,49,55,63,71,77^ HIV/STI intervention,^53,54,61,72^ intimate partner violence,^79,87^ parenting support,^50^ psychological distress,^43,53,65,69,89^ safe partner relationships,^52^ smoking cessation,^44,45,59,68,73,75,76,82,83,88^ substance abuse/misuse,^55,57,62,74,86^ and suicide prevention.^56,58,64,65,67,69,81,84^

#### Type of technology used

**Figure 4.**
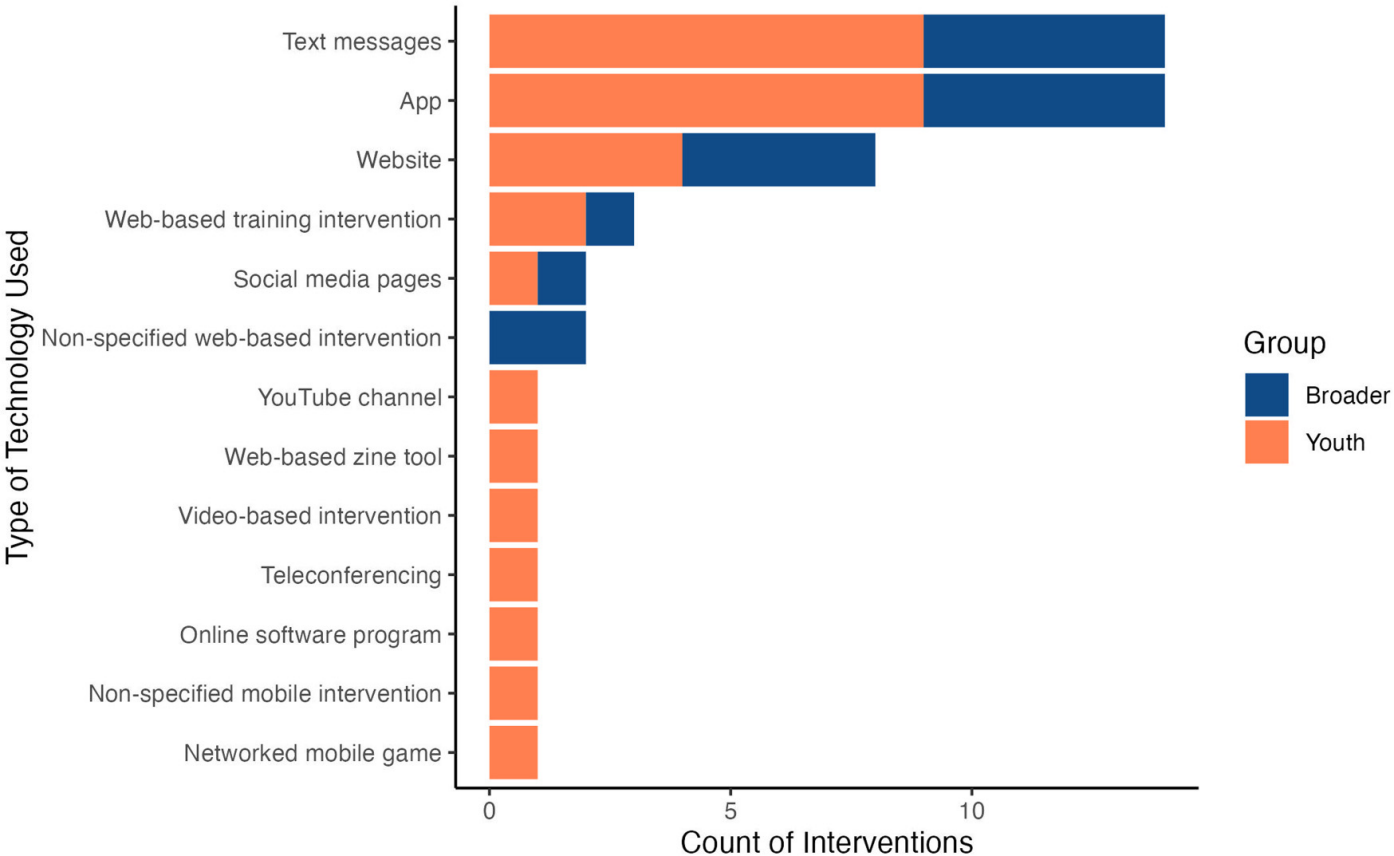
Count of interventions by technology used.

The type of technology used to deliver each e-MH intervention varied across studies. Several studies relied on more than one type of technology so the breakdowns of these studies in this article total more than the total number of studies reported. Counts of the number of interventions using each type of technology are shown in Figure 4. The types of technology used were: apps,^46,48,49,51,52,55,63–65,69,77,83,84,87^ networked mobile games,^70^ non-specified mobile interventions,^66^ non-specified web-based interventions,^74,86^ online software programs,^60^ social media pages,^72,88^ teleconferencing,^71^ text messages,^45,47,50,53,54,58,62,67,72,73,75,76,81,82^ video-based interventions,^61,62^ web-based training interventions,^43,56,85^ web-based zine tools,^68^ websites,^44,57,59,72,78–80,89^ and YouTube channels.^72^ Seven interventions used a multi-media-based approach (four in youth-specific studies and three in broader studies), using combinations of the abovementioned delivery modes. Two studies used both text messages and videos,^62,67^ while others used combinations such as app and text messages,^83^ app and website,^49^ text messages and telephone counseling,^75^ and text messages and websites.^60,72^ One study paired a FitBit-HR Charge 2 with text messages and web-based programs in their intervention.^60^

#### Type of research approaches

**Figure 5.**
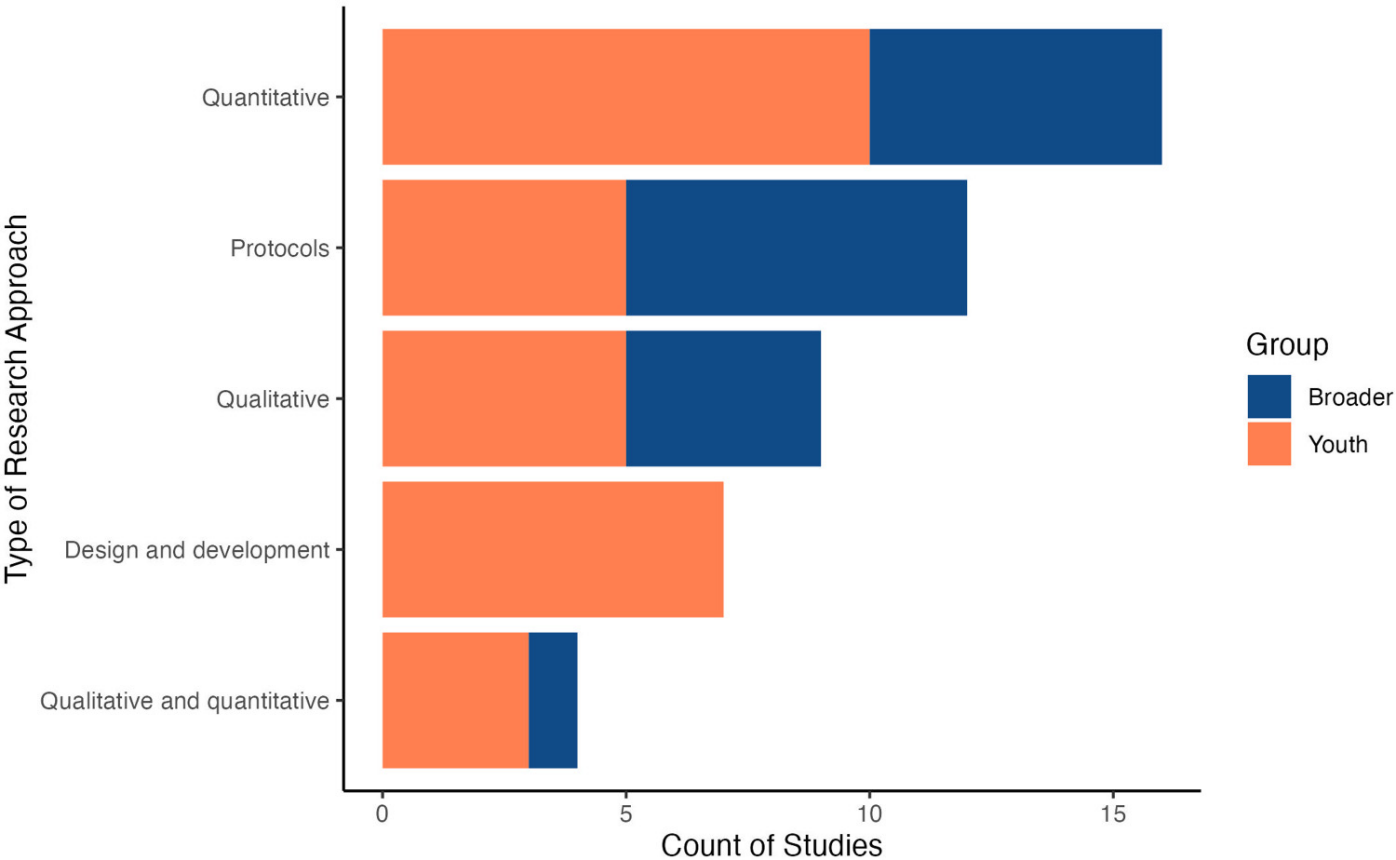
Count of studies by type of research approach.

As noted in the “Introduction” section, the aim of this article is not to thoroughly review or evaluate e-MH intervention outcomes (e.g. effectiveness and efficacy). Therefore, only broad categories of research approaches are reported to gain a sense of the type of research occurring within this area. A wide variety of research approaches were used both youth-specific and broader. Figure 5 provides a visual count of studies by reported research approach. Qualitative approaches were reported in five of the 30 total youth-specific studies,^50–52,68,71^ and in four of the 18 total broader studies,^77,78,84,85^ and assessed outcomes such as user engagement,^50,68^ anticipated benefits,^77^ and cultural relevance.^77,78,84^ Quantitative approaches were reported in 10 youth-specific studies^43–45,47,48,57,58,65,67,72^ and six broader studies^49,76,79–81,89^ and assessed outcomes such as indicators of psychological distress^47,58,65,69^ and substance use.^44,45,47,57^ Some studies reported both qualitative and quantitative approaches (i.e. mixed methods). This occurred in three out of the 30 total youth-specific studies^54,56,69^ and one of the 18 total broader studies.^83^ Other studies reported on the design and development of interventions (rather than reporting qualitative or quantitative approaches) and this occurred for seven out of the 30 total youth-specific studies.^38,46,55,59,61,62,70^ No broader studies shared the same focus of design and development. The main outcome of these design and development studies was often the developed intervention itself,^46,59,61,62,70^ in which assessment of intervention effectiveness is currently ongoing. Finally, study protocols were retrieved for five youth-specific studies,^53,60,63,64,66^ seven broader age-ranged studies,^73–75,82,86–88^ and described the future assessment of outcomes such as measures of psychological distress^53,60,63,64^ and substance use.^73–75,82,86,88^

#### Intervention development

**Figure 6.**
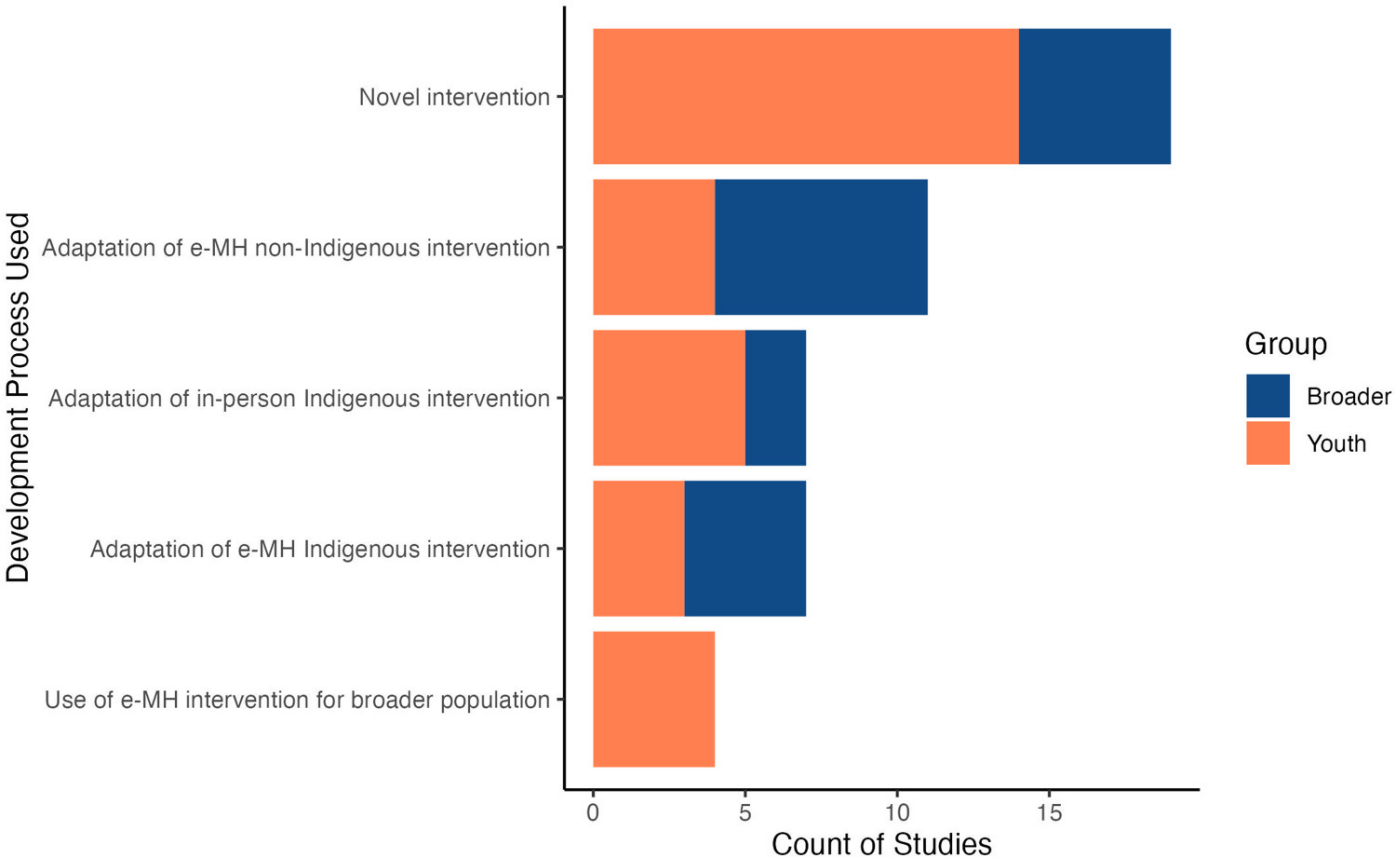
Count of studies by development process used.

Processes for e-MH intervention development and adaptation also ranged widely. Figure 6 provides a visual count of studies by the development process used. Almost half (*n *= 14) of the 30 youth-specific studies developed novel e-MH interventions designed specifically for Indigenous youth/young adults,^45–48,50,53–56,65,69–72^ where as five of the 18 total broader studies developed novel interventions.^49,76,86,88,89^ In contrast, five of the youth-specific studies^51,59,62,66,67^ and two broader studies^75,77^ opted to translate pre-existing in-person interventions developed for Indigenous youth/young adults into e-MH interventions for Indigenous youth/young adults. Four of the youth-specific studies opted to develop e-MH interventions for Indigenous youth/young adults from pre-existing e-MH interventions that had been initially developed for non-Indigenous populations.^44,60,61,68^ Seven of the broader studies did the same.^74,78–81,83,87^ Three youth-specific^38,58,64^ and four of the broader^73,82,84,85^ studies demonstrated iterative development (i.e. development with several opportunities for feedback from future app users, community members, etc.) by adapting pre-existing e-MH interventions designed specifically for Indigenous youth. One youth-specific study examined a pre-existing e-MH intervention that was not specifically adapted to American Indian/Alaskan Native culture but was designed to aid adults who support youth in multicultural settings.^43^ Relatedly, three youth-specific studies described interventions that were co-developed to be inclusive of—but not specifically for—Indigenous youth.^52,57,63^

### Reported facilitators to e-MH intervention implementation

#### Incorporating community-based participatory research approaches

Twenty-eight studies sought participant feedback regarding the design and development of e-MH interventions,^38,46,48–53,55,59,61,62,64,65,67,68,70–72,77,78,81–84,86,87,89^ through processes such as focus groups,^51,59,68,82,87^ co-design workshops,^38^ and think aloud protocols, in which participants tested interventions while being observed and questioned by the researcher.^46,52^ As part of the e-MH intervention research approaches, several studies involved many iterative phases that built upon each other in the co-design process.^38,46,56,59,61,62,68,72,73,77,82^ These processes were used to develop novel interventions^46,56,62^ and adapt previously existing in-person interventions to e-MH interventions.^59,62,77^ Iterative processes were also used to consult, review, and revise previously existing e-MH interventions to become more culturally appropriate^61,68,72,73,82^ or age appropriate^38^ for target participants. Some of these studies included software developers^46^ and media organizations^62^ who supported the development of several drafts of the intervention as well.

Several types of participant groups (e.g. stakeholders and individuals with experience working with AI/AN youth^51^; expert reference groups such as service providers^77^; community advisory boards^70^ and groups^86^) were included in the co-design process. Longer lengths of the research projects (e.g. two years,^81^ three years,^61^ and five years^62^) were highlighted by researchers to allow for the incorporation of a wide array of perspectives and experiences in the intervention design process.^61^ Studies varied in the levels of involvement of participants throughout the study with this process ranging from communities electing to participate in all stages of the study^49^ to others who were only involved in some stages. This level of involvement was also dependent on client variables, such as the level of preparedness to provide critical feedback.^46^

Several studies also noted the importance of establishing and preserving relationships with partnering communities.^50,53,65,67,70–72^ Researchers noted that these partnerships were key to project success^50^ and provided opportunities to demonstrate relational accountability with participants and with governing leadership to make research relevant to the communities represented.^53^ These partnerships enabled engagement through well-established communication channels,^67^ multimedia health resources,^72^ and local partner organizations.^65^

#### Incorporating culture into intervention presentation, content, and study design

Researchers from one study included in this review noted that the “one size fits all” approach is not as effective as approaches that are more culturally matched to their audiences.^68^ Thirty-three studies included in this review described efforts to provide culturally-appropriate interventions for their participants.^38,45,46,48–52,55,59,61,62,64,67,68,70,72–82,84–89^ Some interventions described the inclusion of cultural representation in language and visual aspects of the intervention. For example, culturally relevant text messages,^45,67,72,73,76,81,82^ and the inclusion of culturally relevant language more broadly^51,62,67,68,70,77,78,84,88^ (e.g. through culturally relevant video and auditory components^59,68,84^) were noted. Cultural representation via visual aesthetic choices was also incorporated,^46,77,78,84^ with a few studies including Indigenous media experts, (e.g. a Native-owned film crew *Sky Bear Media*^62^; Indigenous actors^86^; Indigenous artists^64,86^; Indigenous graphic designers^64^) to support participant engagement.

On a more conceptual level, several studies noted the importance of incorporating cultural values^48–50,59,67,70,77^ and cultural norms^67,80^ into interventions. Participants in one study requested that intervention videos not include “romanticized Native imagery,” and instead situations that are relatable to them (e.g. familiar scene locations, and characters that American Indian youth would typically turn to for help^61^). Authors also concluded that the inclusion of Indigenous frameworks in interventions (e.g. Kaupapa Māori model of interaction^52^; Two-Eyed Seeing Framework^55^; and Cultural Variance Framework^88^) were associated with improved user engagement, as was the inclusion of narratives/storytelling^86,88^ and holistic approaches that examined social determinants of Indigenous health.^54,55,67,88^

Beyond intervention content, studies also included culturally tailored aspects in their research design. For example, some studies incorporated culturally tailored assessments (e.g. quizzes,^59^ survey items,^76^ and overall study measures^87^), while others included a comparison arm in the design that was culturally tailored,^59^ or used culturally relevant frameworks while conducting study analyses.^45^ Another study noted cultural considerations in terms of compensation for participants and highlighted their efforts to indigenize the process of accessing federal grant funds for their project (e.g. ensuring that incentive relevance was not questioned at the institutional level; ensuring incentives were received before participation).^55^

#### Enhancing participant engagement

Studies outlined several targeted recruitment methods to support engagement (e.g. via Māori radio station advertising^45^; via e-Newsletters, listservs, conferences, print postcards, tribal epidemiological centers, websites, Native social media influencers, and Native student centers at schools and universities^67^). The use of interfaces that youth were familiar with, such as social media platforms (e.g. Instagram, Snapchat, or TikTok)^38,46,67,88^ and/or web-based-platforms previously used for online classes^59^ was also reported to facilitate youth engagement.

##### Methods of incentivization

Several types of incentivization strategies (e.g. access to a mobile phone and cellular plan^53^; access to additional computer time^44^; and gift cards or monetary reimbursements^67,72,86^) were used to support engagement. Access to requested therapy (e.g. nicotine replacement therapies) and facilitator support were also offered.^59^ With respect to randomized control trial designs, making the intervention be made available to *all* clients interested in the service, rather than just to those who were randomly allocated to the intervention arm was suggested as well.^83^ For example, one study found the use of a pre-randomized controlled design (i.e. Zelen design) to minimize false hopes, resentment, and contamination in the control group participants.^53^ The Zelen pre-randomization design method allowed participants to initially consent to providing observational data and then provided them with an opportunity later for further consent if they were pre-randomized to receive the intervention.

##### Enabling participant autonomy

Interventions that provided participants with opportunities for choice and autonomy also appeared to support participant engagement. One study highlighted that participants were interested in being able to define their own problems and solutions while using an app,^84^ while another highlighted the fact that their app did *not* provide opportunities for personalization as a limitation.^46^ Other studies found that the use of open-ended sharing allowed for participants to set their own health and well-being priorities, and to engage at their own comfort levels.^53,54^ Methodologies that honored participant expertise also supported participant autonomy. For example, one study noted that including Indigenous youth throughout the research process as citizen scientists was a key component for facilitating empowerment,^55^ while other studies discussed involving young participants as researchers and viewing their role as content experts,^50,62^ which provides participants with further autonomy during intervention development.

##### Functional considerations

App use was supported by easy sign-up processes^52^ and clear navigation^52,84^ within apps. Customization of content (e.g. interactive videos)^59^ and of problems/solutions to work on^84^ were also identified as functional considerations that facilitated greater ownership and engagement with app interventions in future iterations of the technology. For text message-based interventions, several studies provided recommendations for message interactivity and frequency.^49,67,83^ Recommendations included requiring more active engagement from users,^49^ using open-ended weekly text messages,^53^ using a “Call to Action” within text messages,^67^ allowing for open-ended sharing options,^75,76^ encouraging anonymous use,^38,84,86^ and using a high message frequency.^83^

#### Developing interventions for those who support youth

Other studies examined interventions that could improve mental health support systems more broadly by increasing support and facilitating skill-building for “natural helpers” (i.e. peers, parents, and trusted adults) within communities.^38^ Online role-play training simulations were used to increase participants' capacity to support youth in psychological distress.^61^ Several interventions also noted providing training and support for mental health workers,^61,71,77,85^ which supported the iterative design process during intervention development^61^ and raised awareness of validated electronic mental health tools that are responsive for target communities.^85^ Additional interventions were used to support frontline workers while in session,^77^ and to support the development of practitioner knowledge and skill via professional-to-professional case consultation.^71^

### Reported barriers to eHealth implementation

#### Limited or lack of resources

##### Funding

Having access to adequate financial resources was described as a barrier to intervention success.^43,62,83,85^ Limited resources were reported to hinder an iterative design process^62^ and lead to changes in the intervention's focus, target population, and intervention strategy over time.^62^ While interventions can be sustainable once developed, unanticipated costs associated with development (e.g. conducting needs analysis, receiving ongoing feedback from subject matter experts and end users, conducting beta testing)^43^ also hindered study success.

##### Access

Several other studies noted access to technology as a limiting factor.^38,49,53–55,67,68,70,76,84,85^ Access to the required technological devices^68,84,85^ (e.g. mobile phones^54^ or devices with proper storage capacity for the intervention^49^) was a noted barrier, as was the fragility of devices used.^77^ Access to technology was noted as a reason that participants decided to opt into a study^76^ and loss of access to technology was a reason that participants opted out.^67^ One^62^ study found that, while several participants in this study did not own a phone, the majority of participants felt that having a mobile phone would be helpful as it would support their access to resources beyond just the presented intervention (i.e. access to health care; access to emotional, mental, and spiritual forms of support during crisis; access to emergency text alerts, such as those for shelter opening during extreme weather or for notifications of “bad batch” incidents; and access to opportunities for privacy).^54^ The authors noted that this lack of phone ownership must be understood within the context of colonization and the intersectional barriers that it has caused (e.g. substance use, poverty, and incarceration).^54^

Difficulty connecting to the Internet or having access to data was also a barrier,^38,49,54,55,59,77,83–85^ particularly in rural and remote areas.^55,59,77^ High costs and existing debt with service providers were reported to prevent participants from maintaining regular cellular connectivity.^53,84^ Suggestions for navigating these barriers included providing mobile data plans for youth,^55^ providing a free, one-time download for required apps,^53,64,84^ and engaging with community organizations that may already have access to the internet (e.g. schools).^55^

#### Intervention scope

e-MH interventions alone may not be sufficient to holistically address user needs,^38,49,52,69,77,83,84^ and could be used in conjunction with other services or supports,^49,52,83,84^ particularly for those with co-morbid or complex mental health needs. Some participants expressed concerns regarding the extent of support that can be obtained from a single app in scenarios where more intensive supports are needed (e.g. ongoing patterns of family violence^52^) or where mental health concerns are caused by broader historical factors, such as colonization.^84^ In one study, individual-focused apps promoting behavioral change had relatively small effects at the population level, suggesting that such options may have a low impact (irrespective of the degree of participation from the target population) in the absence of structural interventions.^49^

#### Technological competence, youth safety, and literacy

Information technology (IT) literacy levels^85^ and technical skill gaps^52^ challenged e-MH intervention use, while support articles^52^ and extensive training with the app^83^ were reported to encourage use. Parents reported concerns maintaining youth safety in an online environment, relating to cyberbullying and inappropriate content or advice on unmonitored boards.^52^ Similarly, addressing aspects of confidentiality, anonymity, and privacy were reported to help maximize engagement.^71^ Literacy and language differences were identified as barriers to intervention use by participants,^84^ while lack of knowledge regarding e-MH interventions (e.g. understanding effectiveness and applicability of resources for specific target populations) was identified as a barrier by healthcare workers.^85^

## Discussion

The aim of this review was to provide an updated summary of the literature on e-MH interventions for Indigenous youth conducted to date. This review shows an increase within this research area as evidenced by the jump from 10 studies previously^37^ to 48 studies presently. This increase brings the need for reflection on facilitators and barriers to implementation processes of e-MH interventions, and for the creation of standards/criteria considerations to support future development.

The most common mental health concerns targeted in studies in this review were smoking cessation and suicide prevention. Text messages, apps, and websites were the most frequently reported types of technology used. Quantitative outcomes were reported most often (i.e. within 16 studies), contrasting the reporting of quantitative outcomes in the previous report (i.e. within three studies).^37^ This review also found more protocol/proposal reports than our previous (12 vs. 2, respectively), supporting the conclusion that the number of studies within this research area is expanding. The inclusion of the “design and development” study outcome category can similarly be taken as evidence for a growing area of research. The most common process for developing an intervention was to create a novel intervention, however, additional development methods were used, such as developing e-MH interventions from previously existing in person interventions for Indigenous youth. Iterative development (i.e. working group consultations, community engagement in treatment adaptations, etc.) was also commonly used, and was noted to be beneficial as it allows for cumulative and targeted efforts to meet the needs of the Indigenous youth the interventions are designed to serve.

Several facilitators were noted to support e-MH intervention development and implementation. Incorporating community-based participatory research approaches throughout was found to be important; namely, including participants in the co-design process,^38,46,48–53,55,59,61,62,64,65,67,68,70–72,77,78,81–84,86,87,89^ ensuring opportunity for an iterative co-design process,^38,46,56,59,61,62,68,72,73,77,82^ and maintaining close partnerships with communities^50,53,65,67,70–72^ were commonly noted features of research designs that supported successful intervention development. Representation of culture was also important to ensure that the e-MH interventions were culturally matched to their Indigenous youth users.^38,45,46,48–52,55,59,61,62,64,67,68,70,72–82,84–89^ This occurred in both surface-level details of the interventions, such as in the language used and images included, and in the deeper level details of the intervention, such as the cultural contexts and values communicated. Including aspects of culture throughout various points in the research design process (e.g. culturally appropriate incentives, assessments, and comparison study arms) also facilitated intervention development.^45,55,59,76,87^

Various methods of motivating participant engagement, such as the use of targeted recruitment methods (e.g. radio station advertisements, word of mouth, and positive-toned Facebook Ads), modern communication mediums (e.g. familiar social networks such as Instagram or TikTok, familiar school websites), and aspects that support participant autonomy (e.g. opportunities for personalization, open-ended sharing, and sharing of participant expertise) also supported intervention development. Functional considerations relating to accessibility (e.g. easy-to-use methods for app navigation and customization) and level of required interactivity (e.g. bi-directional and dynamic messaging) within the e-MH intervention platforms were also important factors considered throughout the development process. Finally, the development of support for those who support Indigenous youth (e.g. community members and primary care providers) was also a reported facilitator.^38,43,61,71,77,85^ This style of approach is valuable because it extends beyond the individual user of e-MH intervention and provides resources that can be used to support Indigenous youth from a community level.

Several barriers were also noted to hinder e-MH intervention development and implementation. Notably, several studies reported having limited access to the resources necessary to conduct their research.^38,43,49,53–55,62,67,68,70,76,83–85^ Insufficient financial resources were commonly reported, as well as insufficient access to the required technological devices and connection services (e.g. cellular networks and Wi-Fi networks). To bypass these barriers, researchers suggested creating apps that only require one-time downloads. Another broad category of barriers found was the limit to the amount of support that can be provided by online interventions.^38,49,52,69,77,83,84^ This limitation was prominent for youth with more severe mental health concerns, suggesting the need for these types of supports to be used in tandem with in-person care. Indigenous youth using e-MH interventions face challenges that are far beyond the scope of a single app (e.g. intergenerational trauma and reconnection to culture) and thus, these supports must be provided in tandem with policies and programs that will also support structural and system-level change. Finally, additional barriers identified included low levels of both IT and general literacy,^49,52,83–85^ as well as aspects of the research designs used (e.g. small samples and short trial durations), which should also be considered when developing and implementing future e-MH interventions.

### Future directions

#### Standards and criteria considerations for future development of e-MH interventions for Indigenous youth

Beyond summarizing study characteristics, facilitators, and barriers, the final purpose of this updated systematic review was to provide considerations for standards and criteria that can be used to inform the processes for e-MH intervention development. The practices that occurred most often in the literature were considered in tandem with the known facilitators and barriers for Indigenous youth accessing e-MH care to generate tangible suggestions for those looking to develop e-MH interventions in the future. The findings of this review are bolstered by several of the recommendations from the previous review.^37^ Our seven considerations for standards and criteria of e-MH intervention development from this review are presented in Table 4.

**Table 4.** Proposed standards and criteria considerations for future e-MH intervention development.

Consideration	Description of consideration and supporting data
1. Include partnering communities at their preferred level of involvement throughout the co-design process	Several studies collected participant feedback throughout the design, development, and testing processes to ensure that the e-MH intervention met the specific needs and preferences of the youth A variety of participants were involved in this process, including youth and community members (e.g. health care workers, family, and elders) who commonly support youth Studies vary in levels of involvement from partnering communities, so communities should be consulted to determine their preferred level of involvement in e-MH intervention development
2. Provide opportunities for iterative feedback	Several studies used feedback to culturally adapt previously existing interventions Building upon iterations of interventions in this way will likely lead to more targeted and collaborative efforts on intervention development, as well as improved relational accountability with target communities
3. Tailor both surface-level (e.g. visuals) and deeper-level (e.g. values) content to youth needs within their current community contexts	Culturally relevant images, videos, language, auditory components, and overall aesthetics were commonly used to make interventions more appealing to young people Culturally relevant contexts and values were also included to better reflect the experiences of the youth and to provide holistic healing mechanisms for Indigenous mental health Several studies involved Indigenous media experts (i.e. Indigenous-owned film crews, Indigenous actors, and Indigenous graphic designers) to bolster youth engagement
4. Design interventions within interfaces that youth are already familiar with	Using pre-existing friendship and social networks (e.g. Instagram, Snapchat, and TikTok) or platforms used to support online learning (e.g. class website) can improve intervention use as youth are already familiar with these contexts Such online platforms likely became more relevant/familiar to youth throughout the COVID-19 pandemic, as they may have been required to shift more socializing and learning online
5. Provide the necessary financial, technological, and organizational resources to complete the intervention	Several studies noted that inadequate funding can be a barrier to intervention success; funding needs for all areas of intervention development must be considered Inadequate access to required technology was also a barrier, so project organizers must ensure that participants have access to both the technological devices and the internet access and/or cellular connectivity required for the intervention Training, support, and organizational policies should also be provided for primary care providers to ensure e-MH intervention use can occur without overburdening the workload
6. Consider e-MH intervention features and aspects of study design that are currently being used to increase engagement	Features of e-MH interventions such as open-ended text messaging and storytelling have previously been used to bolster youth engagement Considerations regarding the technological competence and general literacy of the target group should also be given Aspects of the research design that can be modified to improve engagement should also be considered, such as including relevant and timely incentivization and using culturally appropriate assessments/overall study measures
7. Pair e-MH interventions with in-person, community-based, and societal supports	Several studies noted that e-MH interventions alone may not be sufficient for a variety of reasons (e.g. higher therapeutic needs; needs that require support far beyond the scope of an app) Depending on the target of the e-MH intervention, developers must decide whether their app can be a stand-alone intervention (e.g. preventative care measures) or an intervention that is used adjunctively with additional established treatment options/within the context of broader structural changes Development of support for those who support youth can also be considered during the development of interventions for youth

#### Future reviews needed

To better understand the types of e-MH research that are currently ongoing and if the field is growing (i.e. via the availability of protocol studies), the *categories* of outcomes within studies were briefly summarized. Future systematic reviews should further examine specific e-MH intervention outcomes to better understand the effectiveness of these methods. Specifically, reviewers could examine e-MH intervention effectiveness for apps designed to promote overall well-being among Indigenous youth, which may promote Indigenous views of holistic wellness. Additional review topics within the realm of e-MH interventions may examine which modalities best support user engagement, why developers opt to create novel interventions vs. adapting previously existing ones, and which factors best facilitate the integration of e-MH intervention with additional interpersonal and societal supports.

## Conclusion

There has been an increase in published studies and protocols for eMH interventions used to support the mental wellness of Indigenous youth since our previous examination.^37^ As this research becomes more available, there is a greater need for further summarizing of the types of concerns targeted, technology used, outcomes reported, and processes included for development. Consideration must also be given to the factors that are known to help or hinder youth engagement with these e-MH interventions, and these facilitators and barriers should be addressed in iterative rounds of collaborative research to ensure the specific mental health needs of the targeted population are met. To help meet these needs, we have provided seven considerations relevant to future research examining e-MH interventions designed to support Indigenous youth, which build upon those provided by Toombs et al.^37^ Future research should continue to support the iterative development of e-MH interventions and should aim to fill gaps that currently exist within this area.
